# Tissue plasminogen activator worsens experimental autoimmune encephalomyelitis by complementary actions on lymphoid and myeloid cell responses

**DOI:** 10.1186/s12974-021-02102-5

**Published:** 2021-02-20

**Authors:** Pauline Hélie, Celia Camacho-Toledano, Léonie Lesec, Célia Seillier, Antonio J. Miralles, Maria Cristina Ortega, Sylvaine Guérit, Héloïse Lebas, Isabelle Bardou, Virginia Vila-del Sol, Denis Vivien, Brigitte Le Mauff, Diego Clemente, Fabian Docagne, Olivier Toutirais

**Affiliations:** 1 UNICAEN, INSERM, GIP Cyceron, Institut Blood and Brain @Caen-Normandie (BB@C), UMR-S U1237, Physiopathology and Imaging of Neurological Disorders (PhIND), Normandie Univ, Caen, France; 2 Present address: Theodor Kocher Institute, University of Bern, Freiestrasse 1, CH-3012 Bern, Switzerland; 3 Grupo de Neuroinmuno-Reparación, Hospital Nacional de Parapléjicos, Finca La Peraleda s/n, 45071 Toledo, Spain; 4 Hospital Nacional de Parapléjicos, Finca La Peraleda s/n, 45071 Toledo, Spain; 5 Department of Clinical Research, Caen University Hospital, CHU, Caen, France; 6 Department of Immunology and Immunopathology, Caen University Hospital, CHU, Caen, France

**Keywords:** Tissue plasminogen activator, Neuroinflammation, Experimental autoimmune encephalomyelitis, T cell response, Antigen-presenting cells

## Abstract

**Background:**

Tissue plasminogen activator (tPA) is a serine protease involved in fibrinolysis. It is released by endothelial cells, but also expressed by neurons and glial cells in the central nervous system (CNS). Interestingly, this enzyme also contributes to pathological processes in the CNS such as neuroinflammation by activating microglia and increasing blood–brain barrier permeability. Nevertheless, its role in the control of adaptive and innate immune response remains poorly understood.

**Methods:**

tPA effects on myeloid and lymphoid cell response were studied *in vivo* in the mouse model of multiple sclerosis experimental autoimmune encephalomyelitis and *in vitro* in splenocytes.

**Results:**

tPA^-/-^ animals exhibited less severe experimental autoimmune encephalomyelitis than their wild-type counterparts. This was accompanied by a reduction in both lymphoid and myeloid cell populations in the spinal cord parenchyma. In parallel, tPA increased T cell activation and proliferation, as well as cytokine production by a protease-dependent mechanism and *via* plasmin generation. In addition, tPA directly raised the expression of MHC-II and the co-stimulatory molecules CD80 and CD86 at the surface of dendritic cells and macrophages by a direct action dependent of the activation of epidermal growth factor receptor.

**Conclusions:**

Our study provides new insights into the mechanisms responsible for the harmful functions of tPA in multiple sclerosis and its animal models: tPA promotes the proliferation and activation of both lymphoid and myeloid populations by distinct, though complementary, mechanisms.

**Supplementary Information:**

The online version contains supplementary material available at 10.1186/s12974-021-02102-5.

## Background

Multiple sclerosis (MS) is a chronic disease of the central nervous system (CNS) characterized by lymphoid and myeloid cell infiltration and microglial activation, leading to demyelination and progressive neurodegeneration [1]. Tissue plasminogen activator (tPA), a serine protease involved in fibrinolysis [2], is mainly produced by endothelial cells of vessels [3], whereas this protease is also secreted by several cell types within the CNS such as oligodendrocytes [4], astrocytes [5], or neurons [5, 6]. tPA influences neuroinflammatory and neuroimmune processes [7]. Indeed, its effects are particularly relevant to the context of MS: tPA activity is increased by tenfold in the cerebrospinal fluid (CSF) of MS patients [8], and the protease is found in the perivascular spaces associated to active MS plaques [9].

Myelin oligodendrocyte glycoprotein (MOG)_35-55_-induced experimental autoimmune encephalomyelitis (EAE), a well-established animal model of MS, is characterized by the recruitment and entry of CD4^+^ encephalitogenic T cells into the CNS parenchyma [10]. Interestingly, pioneer studies reported that tPA activity is increased in EAE [11, 12] and that tPA-deficient mice (tPA^-/-^) declare EAE later than their wild-type (WT) counterparts, with less severe symptoms in the early phase of the disease, reduced demyelination, axonal damage, microglial activation, and T cell presence in the parenchyma [11]. Later studies in slightly different experimental designs drew opposite conclusions, showing earlier and more severe disease in tPA^-/-^ mice [13]. This indicates that tPA can provide different effects in the EAE course, depending on the phase of the disease and the experimental design.

Several actions of tPA can participate in its controversial effects in EAE. In addition to its protease activity, tPA exerts various functions due to its five distinct functional domains: finger domain, epidermal growth factor (EGF)-like domain, kringle 1, kringle 2, and protease domain [14]. We have previously shown that tPA, by activating the EGF receptor (EGFR), has an anti-apoptotic effect over oligodendrocytes [4] and a chemotactic effect on their progenitors that helps remyelination after chemically induced white matter lesions [15]. tPA is also able to increase blood–brain barrier (BBB) permeability and helps leukocyte diapedesis [16] by several mechanisms, including the potentiation of endothelial N-methyl-D-aspartate receptors (NMDAR) [17] or tight junction disruption by the modification of occludin phosphorylation [18]. Our group has shown that Glunomab®, a monoclonal antibody which prevents the binding of tPA on the GluN1 subunit of NMDAR [19], inhibits leukocyte entry into the CNS, thus blocking demyelination and EAE progression [17].

In addition to these actions, controversial data indicate that tPA may directly act on cells of the innate immunity. tPA can regulate myeloid cell functions, but the pro- or anti-inflammatory nature of this action is still a matter of debate [20, 21]. On the other hand, the possible role of tPA on lymphoid cell activity is still poorly understood [7]. Therefore, the purpose of this study is to investigate the respective actions of tPA on lymphoid and myeloid cells in the context of EAE. Interestingly, we report a deleterious role of tPA in EAE, that may be due to an increase in T cell proliferation, combined to a direct potentiation of dendritic and macrophage antigen presenting function. These combined effects of tPA converge to the promotion of pro-inflammatory T cell adaptive immune response in EAE.

## Methods

### Animals

Transgenic tPA^-/-^ (C57BL/129 background) [22] and C57BL/129 control mice, aged 8–12 weeks, were provided by the *Centre Universitaire de Ressources Biologiques* (CURB, University of Caen, France). C57BL6/J mice aged 6–12 weeks were obtained from Janvier Laboratories. Mice were housed in our local conventional animal facilities. All procedures were performed according to the guidelines of the institutional ethics committee (*Comité Normand d’éthique en matière d’expérimentation animale* (CeNomExa) and *Comité Ético de Experimentación Animal del Hospital Nacional de Parapléjicos*). This was submitted to these committees for approval in accordance with the European directive no. 2013/63/UE (agreement number D14118001) and with the French and Spanish National and Regional Guidelines for Animal Experimentation and the Use of Genetically Modified Organisms (French Ministry of Research, project license number 02653.2, Decree 87/848; RD 53/2013 and 178/2004, Law 32/2007 and 9/2003, Decree 320/2010).

### Reagents

Recombinant human tPA (Actilyse ®) was purchased from Boehringer Ingelheim (Ingelheim am Rhein, Germany). Several controls of tPA and other reagents were used along the study: dialyzed tPA, tPA GGACK, or corresponding vehicles (DMSO, Sigma-Aldrich). tPA dialysis was performed for 48 h at 4 °C against 0.3 M HEPES to remove the arginine vehicle. tPA GGACK was obtained by incubating GGACK (1,5-dansyl-L-glutamyl-L-glycyl-L-arginine chloromethylketone, EMD) with Actilyse in a fourfold molar excess for 6 h at room temperature (RT), followed by dialysis for 48 h at 4 °C with 0.3 M HEPES to remove unbound GGACK and the arginine vehicle. The loss of proteolytic activity of tPA GGACK was confirmed with spectrozyme assay (American Diagnostica) [4]. Aprotinin and epsilon-aminocaproic acid (ε-ACA) were purchased from Sigma-Aldrich. AG1478 hydrochloride was purchased from Tocris. Antibodies used in this study are listed in Suppl. Table 1.

### EAE induction

Monophasic EAE was induced in 6–12-week-old C57BL6/J female and C57BL/129 tPA^-/-^ male mice by active immunization with MOG_35-55_ peptide (Cambridge Research Biochemicals, Genscript). To this end, mice were injected subcutaneously with 200 μg of Complete Freund’s adjuvant (Sigma-Aldrich) containing 400 μg of inactivated particles of *Mycobacterium tuberculosis* (BD Biosciences). The emulsion was administered to regions above the shoulders and the flanks into four sites (50 μL at each injection site). All animals were intraperitoneally injected at days 0 and 2 with 250 ng of pertussis toxin (Sigma-Aldrich) in 200 μL of saline. Mice were weighted and scored daily in a double blind manner for clinical signs of EAE as follows: 0, no disease; 1, limp tail; 2, hindlimb weakness/ no hindlimb reflex; 3, hindlimb paresis; 4, hindlimb paralysis; 5, moribund or dead. In accordance with the ethical standards and regulations, the humane endpoint criteria were applied when an animal reached a clinical score of ≥ 4 for more than 48 h or presented signs of stress or pain (generation of sounds, stereotypic behavior, lordokyphosis, hair loss, or loss of weight superior to 2 g/day for more than 48 h).

### Isolation of leukocytes from spleens and spinal cords

Mice were deeply anesthetized with 5% isoflurane (Aerrane, Baxter) and transcardially perfused with 50 mL of cold phosphate-buffered saline, pH 7.4 (PBS, Sigma-Aldrich). Spinal cords were harvested at the EAE plateau phase [day post-immunization (dpi) 20 ± 3] and homogenized in PBS. Leukocytes were recovered at the 30:70% Percoll (Fisher Scientific) interface after gradient centrifugation as described in the literature [23] and were then counted with the Malassez chamber. Spleens were aseptically removed from naïve and MOG-immunized C57BL/6 mice at the peak of clinical score (≥ 3, dpi 15–18), as described previously [24], mechanically processed to obtain a splenocyte suspension by passing the cells through a 40-μm filter (Falcon). Erythrocytes were lysed in lysis buffer [0.15 M NH4Cl, 9 mM HKCO3, 0.5 M EDTA, pH 7.4 (Stemcell Technologies)], and the sterile splenocytes were resuspended in supplemented sterile PBS with 10% filtered and inactivated fetal bovine serum (FBS, Stemcell Technologies), 1% Penicillin/Streptomycin (Gibco), and 2.5% (v/v) HEPES (Fisher).

### Analysis of the effect of tPA on myeloid cell functions

Splenocytes (10^6^ per well in 24 well plates) were cultured and maintained in RPMI (Gibco) supplemented with 10% FBS (Linus), 1% Penicillin/Streptomycin, 2.5% (v/v) HEPES, 2 mM L-glutamine (Gibco), and 50 μM β-mercaptoethanol in the presence of the following molecules for 24 h: tPA (0.2, 2, and 20 μg/mL) or its vehicle [34.84 mg/mL arginine (Sigma-Aldrich), 10.72 mg/mL phosphoric acid (Sigma-Aldrich) and 0.1 mg/mL Tween 80 (Sigma-Aldrich)], Glunomab® antibody (10 μg/mL) or its isotypic control [17], 2 μg/mL tPA-GGACK (with blocked serine protease activity), 5 μM of the EGFR inhibitor (AG1478 hydrochloride, additional 30-min preincubation).

### Analysis of the effect of tPA on lymphoid cell proliferation

Naive splenocytes (1.5 × 10^6^/mL in suspension) were incubated for 20 min at RT in PBS with 0.5 μM CFSE (Life technologies). Cells were then washed and suspended in complete DMEM containing: 1% penicillin–streptomycin (P/S, Sigma-Aldrich), 1% GlutaMax-I 100X (Gibco), 10% FBS, and 0.1% β-mercaptoethanol. Ninety six-well U-bottom plates were coated with 1 μg/mL anti-mouse CD3 antibody (eBioscience) in PBS overnight. The plates were washed twice with 200 μL of PBS and 10^5^ labelled splenocytes were plated (P96 well) in 200 μl of complete DMEM and incubated for 96 h with 1 μg/mL anti-mouse CD28 antibody (eBioscience) in the presence or not of tPA at different concentrations (0.1, 1, and 10 μg/ml [4], 10 μg/ml Glunomab® antibody or its isotypic control [17], ε-ACA, an inhibitor of the protease function of tPA (200 mM), the plasmin inhibitor aprotinin (20 IU/mL^−1^) [25], or tPA-GGACK (10 μg/mL). Then, cells were harvested by centrifugation (2000 rpm, 10 min, RT), washed in PBS and stained for the flow cytometry assay. To study the effect of tPA during MOG-induced stimulation, splenocytes were obtained from MOG-immunized C57BL6/J mice at the peak of clinical score (≥ 3), as described previously [24, 26] and were plated in IMDM (BioWest, Nuaillé, France), supplemented with 2-mM L-glutamine, 1% P/S, 10% FBS (Gibco), and 50-μM β-mercaptoethanol, in U-bottom 96-well plates at a density of 2 × 10^5^ cells. Splenocytes were exposed to 2-μM Tag-it Violet^TM^ Proliferation and Cell Tracking Dye (Biolegend) diluted in PBS supplemented with 0.1% BSA at 37 °C with shaking for 20 min, protected from light. After washing, splenocytes were stimulated for 72 h with 5 μg/mL MOG and treated with 2 μg/mL tPA, 5 μM AG1478 or the combination of both. Cells were harvested by centrifugation at 2000 rpm at RT, washed in PBS and stained for the flow cytometry assay.

In order to analyze the direct effect of tPA on MHC-II upregulation and on T cell proliferation, splenocytes from EAE mice at the peak of their clinical symptoms were depleted of CD3 T cells by cell sorting in a FACS Aria IIu. These splenocytes were plated at 2 × 10^5^ cells per well in U-bottom 96-well plates with and were exposed to MOG or MOG + tPA. After 24 h, the medium was replenished in all culture conditions, and 5 × 10^4^ sorted Tag-it Violet-labelled CD3^**+**^ T cells from other EAE mice at the peak of their clinical symptoms were added to each well. In one of the culture conditions, tPA was removed to check the direct effect of the protease on antigen presentation capacity. After 72 h, cells were harvested, and CD3, CD4, and CD8 proliferative cells were counted in a FACS Canto II cytometer.

The proliferation index has been calculated dividing the sum of the cells in all generations by the computed number of original parent cells, which were theoretically present at the beginning of the experiment. Analysis of data was performed using the ModFit LT™ software (Verity Software House).

### Flow cytometry

Cells were resuspended in 50 μl of staining buffer, and Fc receptors were blocked for 15 min at 4 °C with 10 μg/mL anti-CD16/CD32 antibodies (BD Biosciences.) After the blocking step, cells were labelled for 20 min at 4 °C with corresponding fluorochrome-conjugated monoclonal antibodies (Suppl. Table 1). The BD Pharmingen transcription factor buffer set was used according to the manufacturer’s protocol to detect expression of FoxP3. When necessary, cells were fixed in a final volume of 300 μl with 4% paraformaldehyde (PFA) for 10 min. For detection of intracellular cytokines, splenocytes were stimulated for 5 h with 500 ng/mL PMA (Sigma-Aldrich) and 500 ng/mL ionomycin (Sigma-Aldrich) in the presence of 10 μg/ml brefeldin A (Biolegend). Then, cells were fixed and permeabilized with cyto Fix/Perm (Biolegend) and stained at RT for 30 min. Samples were acquired on a FACS Verse or a FACS Canto II cytometer (Beckton Dickinson), and data were analyzed with the FlowJo 7.6.5 software (TreeStar Inc.). Cell frequencies were determined by flow cytometry and absolute numbers calculated from counts determined using a hemocytometer (Malassez chamber).

### Cytokine assay

Levels of cytokines in supernatants of anti-CD3/CD28 stimulated splenocytes were determined using the cytometric bead array (CBA) mouse Th1/Th2/Th17 cytokine kit (BD Biosciences) according to the manufacturer’s protocol. Measurements were performed using FACS Verse flow cytometer, and data were analysed with the FCAP Array^TM^ software (version 3.0).

### Immunofluorescence

Mice at the EAE plateau phase (d20 ± 3) and with matching mean scores were deeply anesthetized and transcardially perfused with 50 mL of cold 1X PBS. Tissue samples were postfixed for 24 h at 4 °C with 4% PFA and then cryoprotected for 24 h at 4 °C with 20% sucrose solution, before freezing process into Tissue-Tek (Miles Scientific). Samples were then cut with a cryomicrotome (Leica) to obtain 10-μm sections which were thaw-mounted on polylysine-coated slides and stored at – 80 °C before experiment. After warming, sections were rehydrated three times in PBS during 15 min and incubated overnight at RT with the following antibodies: rabbit anti-CD3 (1:200, Abcam ab5690), rat anti-CD4 (1:25, eBiosciences 14-0042-82, clone RM4-5), and goat anti-colIV (1:1000, Southern Biotech 1340). Primary antibodies were revealed by using affinipure F(ab’)2 fragments of donkey IgGs conjugated to Alexa 647, Alexa 488 or Cy3 (1:1000, Jackson Immuno Research). Then, sections were washed in PBS and coverslipped with antifading medium containing DAPI. Images were digitally captured on Leica DM6000 microscope-coupled coolsnap camera, visualized with Metavue 5.0 software (Molecular Devices, USA) and further processed using ImageJ 1.49e software (NIH). For cell counting, images of three WT and three tPA^-/-^ hemi-spinal cord sections from cervical, upper thoracic, lower thoracic, and lumbar/sacral regions were analyzed with the Fiji (ImageJ) software: an image-by-image threshold was applied to obtain a binary image for automatic particle counting (cells/mm^2^) using a minimal particle size assigned to overcome the background noise of the image.

### Statistical analysis

Results are presented as the mean + SEM and were analysed with the GraphPad Prism and SigmaPlot 11.0 softwares. Normality tests were performed on all samples (Agostino-Pearson omnibus and Shapiro–Wilk tests). Nonparametric Kruskal–Wallis for multiple comparisons were used, followed by two-by-two comparisons by Mann–Whitney’s U tests when relevant. For multiple comparisons, an ANOVA test or its corresponding ANOVA on RANKS was performed followed by the Tukey or Dunn post hoc tests (compared with the control condition or all pairwise), respectively. The minimum value of statistical significance considered was *P* < 0.05; for comparison of incidence curves, log-rank (Mantel-cox test) was used.

## Results

### EAE is less severe in tPA-deficient mice

**Fig. 1:**
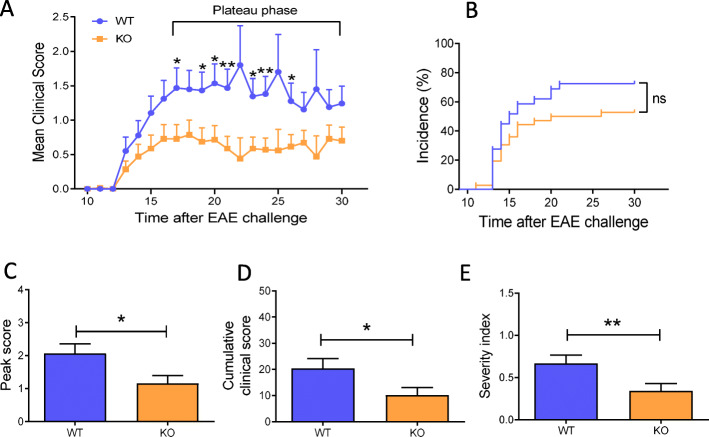
tPA^-/-^ mice show less EAE symptoms than WT mice. **a** Mean EAE clinical score, **b** incidence, **c** peak score, **d** cumulative clinical score, and **e** severity index for WT and tPA^**-/-**^ mice. Results are expressed as mean + SEM. *N* = 29 (WT) and *N* = 35 (tPA^-/-^). ^*^P < 0.05 and ^**^P < 0.01

tPA^-/-^ mice developed less severe EAE disease than their WT counterparts (control group), with lower clinical scores during the plateau phase between dpi 17 and 23 (d20 ± 3) (Fig. 1a). Although no significant difference was observed between tPA^-/-^ and WT mice in the incidence of the disease (*P* = 0.1194, Fig. 1b) nor for the day of disease onset (tPA^-/-^ 15.13 ± 0.88 *vs* WT 14.6 ± 0.45, *P* = 0.8159, data not shown), peak score and cumulative clinical score were significantly lower in tPA^-/-^ than in WT mice (tPA^-/-^ peak score 1.16 ± 0.23 *vs* WT 2.07 ± 0.29, *P* = 0.0164; cumulative clinical score 10.17 ± 2.89 *vs* 20.38 ± 3.76, *P* = 0.0163, Fig. 1c and d). In addition, severity index ([26]), was significantly lower in tPA^-/-^ than in WT mice (tPA^-/-^ severity index 0.3440 ± 0.08 *vs* WT 0.6652 ± 0.10, *P* = 0.0075, Fig 1e).

### tPA enhances T cell response by a proteolytic mechanism

**Fig. 2:**
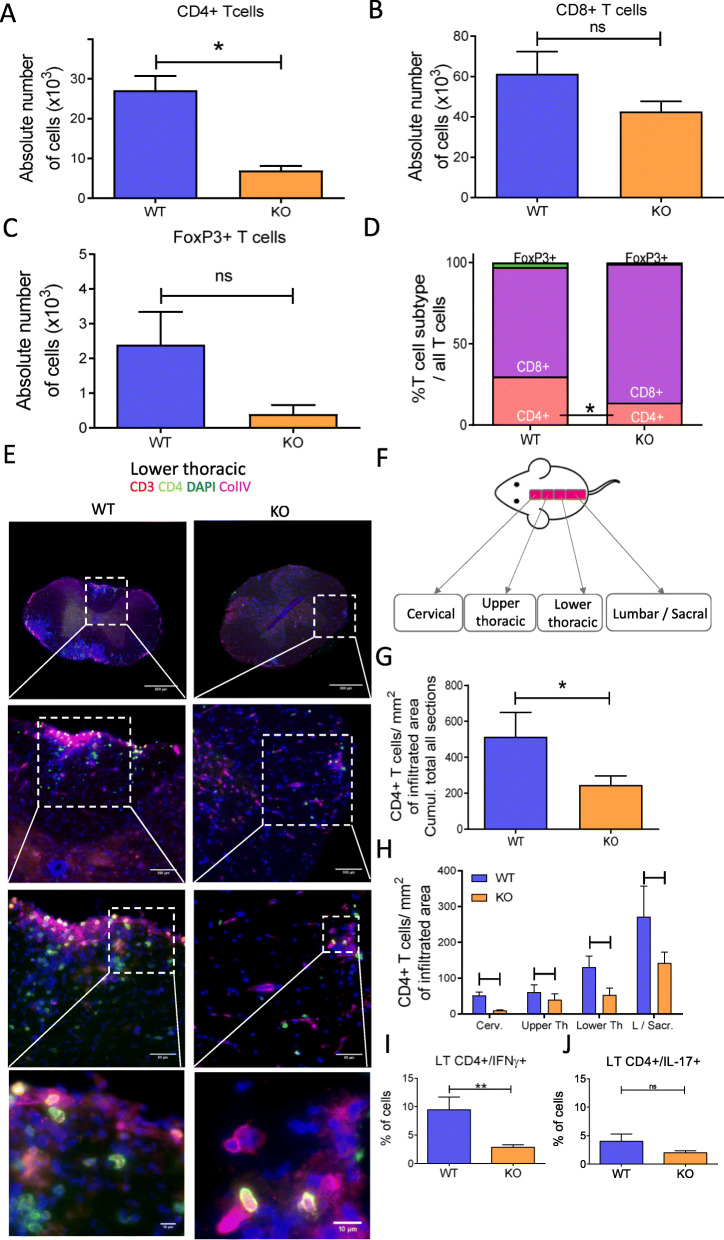
tPA^-/-^ mice show reduced CD4^+^ T cell infiltration than WT in the spinal cord. Absolute number of infiltrated **a** CD4^+^, **b** CD8^+^, and **c** FoxP3^+^ T cells in spinal cords of WT and tPA^**-/-**^ EAE mice measured by Malassez cell counting and flow cytometry. Results are expressed as mean + SEM. *N* = 6 (WT) and *N* = 3 (tPA^-/-^). ^*^P < 0.05. **d** Percentages of T cell subtypes relative to total CD3^+^ T cell number in the spinal cord of tPA^-/-^ and WT EAE mice. **e** Photomicrographs show representative immunofluorescence staining (from *N* = 3 for tPA^-/-^and WT) for CD3, CD4, and COLIV markers in the lower thoracic region of mice spinal cords. Nuclei were counterstained with DAPI (blue). **f** Scheme describing the different parts of the spinal cord. **g** and **h** Quantification of the average density of CD4^+^ T cells within infiltrated areas in **g** the whole spinal cord and in **h** individual segments (*Cerv.* cervical, *Upper Th.* upper thoracic, *Lower Th.* lower thoracic, *L/Sacr.* lumbar/sacral. (*N* = 3). ^*^P < 0.05. **i** and **j** Intracytoplasmic cytokine detection by flow cytometry in splenocytes from tPA^-/-^ and WT EAE mice (10 days post-immunization) after stimulation with PMA/ionomycin. Graphs show the percentage of CD4^+^/IFN-γ^+^ T cells and CD4^+^/IL-17^+^ T cells. *N* = 7 (WT) and *N* = 7 (tPA^-/-^). ^**^P < 0.01

Given that immune cell infiltration is a cardinal feature of EAE, we analyzed T cell subpopulations in the spinal cords of EAE mice during the plateau phase of the disease (d20 ± 3) by flow cytometry (Fig. 2 and Suppl. Fig. 1) and immunofluorescence (Suppl. Fig 2). We found that the absolute number of CD4^+^ T cells was lower in tPA^-/-^ than in WT mice (6940 ± 1220 *vs* 27,110 ± 3642 cells; *P* = 0.0238, Suppl. Fig 1B, Fig. 2a), an observation consistent with the clinical scores observed in tPA^-/-^ EAE mice (Fig. 1). Neither CD8^+^ (WT 61,291 ± 11,070 cells *vs* tPA^-/-^ 42,721 ± 4988 cells, *P* = 0.5476, Fig. 2b) nor regulatory FoxP3^+^ T cell (T_reg_) (WT 2396 ± 2322 cells *vs* tPA^-/-^ 394.6 ± 444 cells; *P* = 0.1667, Fig. 2c) were significantly altered. In accordance with the above data, CD4^+^ T cells were the only CD3^+^ T cell subset that showed changes in the spinal cord of tPA^-/-^ mice (tPA^-/-^ 13.87% ± 2.44 *vs* WT 29.85% ± 4.01, *P* = 0.0476, Fig. 2d). Concordantly, the histological analysis of the spinal cords (Fig. 2e) further showed that the average density of CD4^+^ T cells within infiltrated area was lower in tPA^-/-^ than in WT EAE mice (absolute number of WT 514.4 ± 135.7 *vs* tPA^-/-^ 245.3 ± 50.78, *P* = 0.0495, Fig. 2g and Suppl. Fig. 2A-C) and that these changes were mainly due to differences in the cervical region (WT 51.33 ± 13.49 vs tPA^-/-^ 9.97 ± 1.98, *P* = 0.0495, Fig. 2h). Interestingly, when analyzing the cytokine profile of CD4^+^ T cells *in vivo*, we showed that the percentage of IFN-γ/CD4^+^ T cells—but not IL-17/CD4^+^ T cells—was significantly decreased in tPA^-/-^ EAE mice as compared with WT EAE (IFN-γ/CD4^+^ T cells WT 9.4% ± 4 vs. tPA^-/-^ 2.8% ± 0.8, *P* = 0.011; IL-17/CD4^+^ T cells WT 4% ± 2.3 vs. tPA^-/-^ 2% ± 0.6, *P* = 0.3; Fig. 2i and j, Suppl. Fig 1C).

**Fig. 3:**
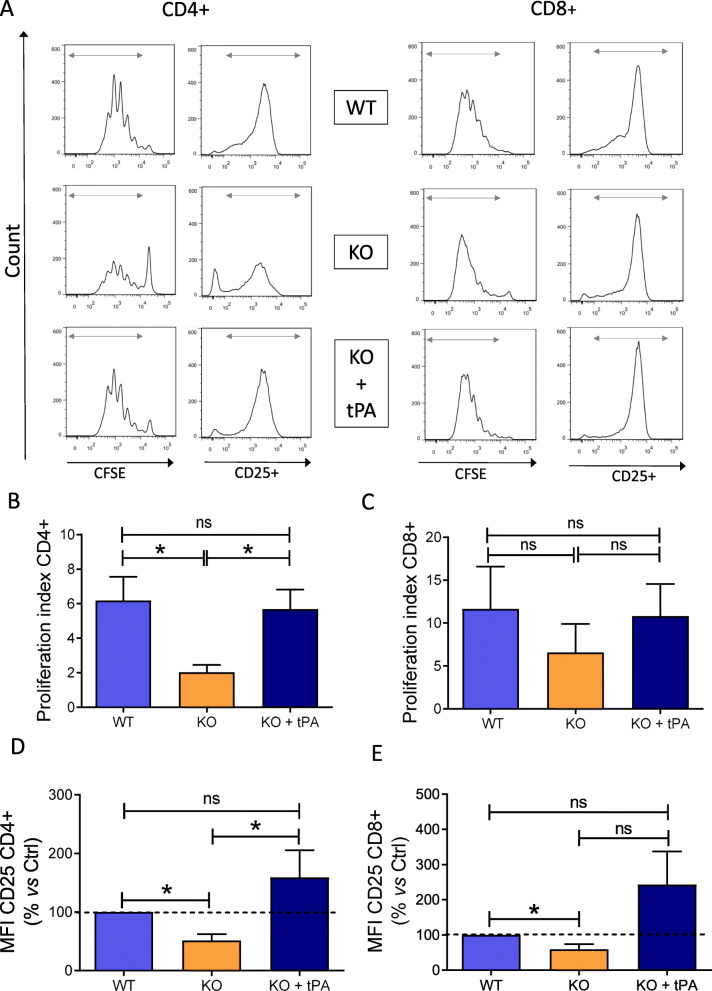
Endogenous tPA stimulates CD4^+^ T cell proliferation and activation. tPA^-/-^ and WT splenocytes were activated with anti-CD3 and anti-CD28 antibodies (both 1 μg/mL) and treated in the indicated conditions for 4 days. **a** Representative flow cytometry histograms for proliferation (CFSE) and activation (CD25^+^) of T cells. Corresponding quantification of proliferation index for **b** CD4^+^and **c** CD8^+^ T cells. Percentage of increase of CD25 for **d** CD4^+^ and **e** CD8^+^ T cells. Results are expressed as mean + SEM (*N* = 5). ^*^P < 0.05

To determine the mechanisms involved in the decrease of CD4^+^ T cell number in tPA^-/-^ EAE mice, we assessed their functional response *in vitro* after activation with anti-CD3ε/CD28. CD4^+^ T cells from tPA^-/-^ naive mice proliferated less than CD4^+^ T cells from their WT counterparts (proliferation index: tPA^-/-^ 2.03 ± 0.43 *vs* WT 6.18 ± 1.38; *P* = 0.0317; Fig. 3a and b). Activation level measured by mean fluorescence intensity (MFI) of CD25 was also reduced in CD4^+^ T cells from tPA^-/-^ as compared with WT CD4^+^ T cells (51.42% ± 10.96 of WT; *P* = 0.0286, Fig. 3d). Importantly, the addition of exogenous tPA rescued the proliferation and activation of tPA^-/-^ CD4^+^ T cells at the same level as CD4^+^ T cells from WT counterparts (*P* = 0.8413 and *P* = 0.3143, respectively; Fig. 3a, b, and d). As in the case of the *in vivo* analysis, CD8^+^ T cell proliferation was not affected in tPA^-/-^ (proliferation index 11.65 ± 4.93 *vs* tPA^-/-^ 6.57 ± 3.35, *P* = 0.3095) although their activation was reduced (59.34% ± 14.48 of WT, *P* = 0.0286; Fig. 3a, c and e).

**Fig. 4:**
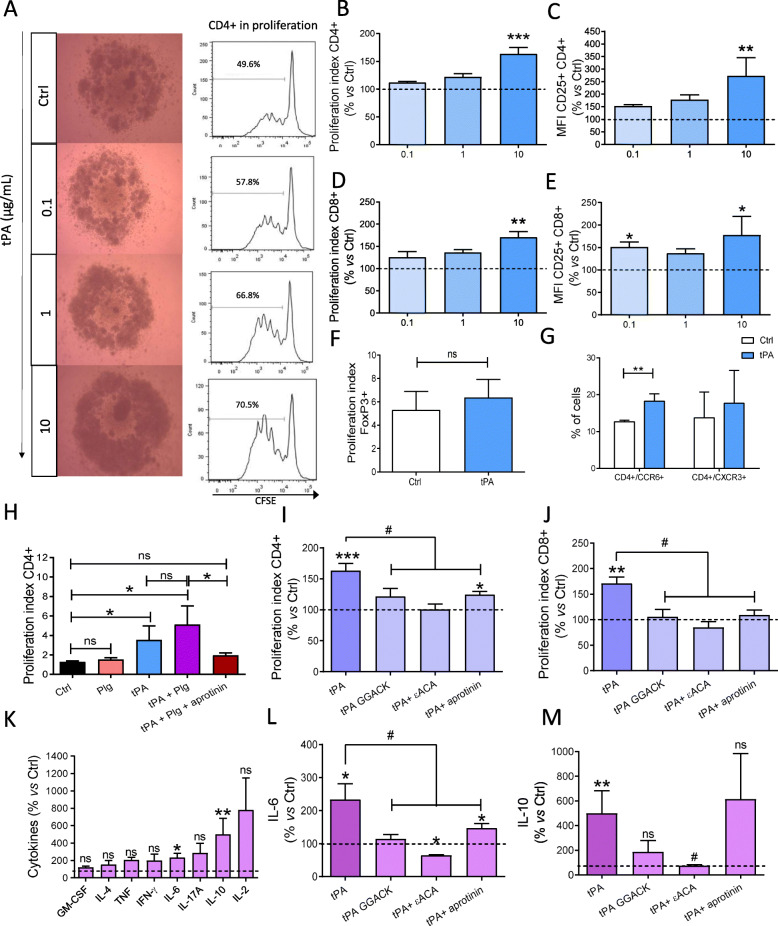
tPA activates CD4^+^ and CD8^+^, but not FoxP3^+^ T cells *in vitro* by a proteolytic mechanism. Splenocytes from naive mice were activated with anti-CD3 and anti-CD28 antibodies (both 1 μg/m) and treated in the indicated conditions for 4 days. **a** Proliferation estimated by observation under bright field binocular (left) and representative flow cytometry histograms for CFSE fluorescence (right), indicating the number of cells in proliferation state at the time of the experiment. Quantification of proliferation index (CFSE) and CD25^+^ MFI (as index of activation) for **b, c** CD4^+^ and **d, e** CD8^+^ (*N* = 4–9). **f** Quantification of proliferation index for FoxP3^+^ T cells (*N* = 3). Proliferation index are expressed as mean + SEM percentage *vs* control (Ctrl = 100% baseline). Activation is expressed as mean + SEM percentage of increase of CD25 MFI. **g** Quantification of CD4^+^/CCR6^+^ T and CD4^+^/CXCR3^+^ T cells (*N* = 4). **b–g** ^*^P < 0.05, ^**^P < 0.01, ^***^P < 0.001 *vs* control. **h–j** Proliferation index of CD4^+^ and CD8^+^ T cells in the indicated treatment conditions. *N* = 5–9; ^*^P < 0.05, ^**^P < 0.01, ^***^P < 0.001 *vs* control; ^#^*P* < 0.05 *vs* tPA. **k, l** Cytokine measurements (percentage of control) in activated splenocytes treated in the indicated conditions (*N* = 4–7). ^*^P < 0.05, ^**^P < 0.01 *vs* control; ^#^*P* < 0.05 *vs* tPA

We next examined the effect of tPA on T cell response *in vitro*. tPA significantly increased proliferation and activation of anti-CD3ε/CD28 activated CD4^+^ and CD8^+^ T cells in a dose-dependent manner, in comparison with the control group of untreated cells (Fig. 4a-e). At the dose of 10 μg/mL of tPA, proliferation of CD4^+^ and CD8^+^ T cells were increased to reach 163.40% ± 11.28 (*P* = 0.0004) and 170.90% ± 12.59 (*P* = 0.0012), respectively. No effect of tPA was observed on T_reg_ cells (*P* = 0.9999, Fig. 4f). In order to further analyze the profile of CD4^+^ T cells involved in the response to tPA treatment, we analyzed by flow cytometry the expression of CCR6 and CXCR3, two chemokine receptors involved in the CNS trafficking of Th17 and Th1 cells, respectively. We observed an increase in the percentage of CCR6^+^/CD4^+^ T cells, with no changes in the percentage of CXCR3^+^/CD4^+^ T cells after treatment of splenocytes with tPA (10 μg/mL) for 72 h (Fig. 4g).

Next, we investigated the mechanisms underlying tPA actions on T cells. Since the primary action of tPA is to activate plasminogen into plasmin, we interrogated the different elements of the tPA/plasminogen/plasmin cascade. Plasminogen treatment neither increased CD4^+^ T cell proliferation nor potentiated the proliferative action of tPA on CD4^+^ T cells (*P* = 0.400 and *P* = 0.200; Fig. 4h). However, aprotinin, a specific inhibitor of plasmin, did reverse tPA-mediated activation (for CD4^+^ T cells, *P =* 0.0190*;* for CD8^+^, *P =* 0.0109; Fig. 4i and j). In addition, the inactivation of the catalytic activity of tPA (tPA-GGACK) abolished tPA stimulatory effect on CD4^+^ and CD8^+^ T cell proliferation (*P =* 0.0252 and 0.0162, respectively; Fig. 4i and j). In addition, ε-ACA, an inhibitor of plasmin generation from plasminogen inhibited the stimulatory effect of tPA on CD4^+^ and CD8^+^ T cell proliferation (*P* = 0.0056 and *P* = 0.0040, respectively; Fig. 4i and j). This indicates that tPA increases T cell proliferation *via* the proteolytic activation of plasminogen into plasmin.

We next explored if tPA-mediated effects on T cell proliferation was related to its previously demonstrated proteolytic action on NMDAR [27], as this receptor was previously reported to be expressed on T cells [28, 29]. Glunomab®, a monoclonal antibody that blocks the interaction between tPA and NMDAR [19], did not alter the proliferative effect of tPA on CD4^+^ T cells (Supplementary Fig. 3). This result excluded that tPA may act on T cell proliferation by acting on NMDAR.

Furthermore, since cytokines are key mediators of T cell-driven autoimmunity, we analyzed the impact of tPA on the late cytokine pattern of activated T cells. tPA-induced an increase of IL-6 and IL-10 secretion by activated splenocytes at 4 days of culture (respectively 233.9% ± 47.45, *P* = 0.0289 and 499.4% ± 184.3, *P* = 0.0029, Fig. 4k). In addition, concerning IL-6, this effect was not observed with tPA-GGACK and was inhibited in the presence of ε-ACA or aprotinin (*P* = 0.0167, 0.0167, and 0.0333, respectively, Fig. 4l). ε-ACA also reverted the activation of IL-10 secretion by tPA (*P =* 0.0167, Fig. 4m). Together, these data indicate that tPA increases T cell proliferation *via* the generation of plasmin to increase their proliferation, activation, and secretion of cytokines.

### tPA enhances myeloid cell maturation by the activation of EGFR

**Fig. 5:**
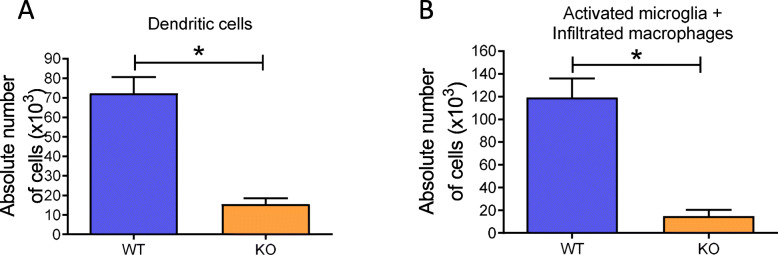
tPA^-/-^ mice show reduced myeloid cell infiltration than WT in the spinal cord. Absolute number of infiltrated **a** DCs and **b** Mɸ^+^ activated microglia in half-spinal cords of WT and KO EAE mice measured by Malassez cell counting and flow cytometry. Results are expressed as mean + SEM [*N* = 6 (WT) and *N* = 3 (tPA^-/-^)]. ^*^P < 0.05

Our next step was to analyze whether the distribution of myeloid cells is altered in the spinal cord of tPA^-/-^ EAE mice. We found that absolute numbers of CD11c^+^/CD11b^+^ (dendritic cells, DCs) and CD45^high^/CD11c^-^/CD11b^high^ (activated microglia and infiltrated macrophages, Mɸ) were lower in the spinal cords of tPA^-/-^ EAE mice as compared with their WT EAE counterparts (DCs 15,474 ± 2984 *vs* 72,283 ± 8330; *P* = 0.0238; microglia/Mɸ: 14,980 ± 5442 *vs* 119,228 ± 16,810, *P* = 0.0238; Suppl. Fig 4, Fig. 5a and b).

**Fig. 6:**
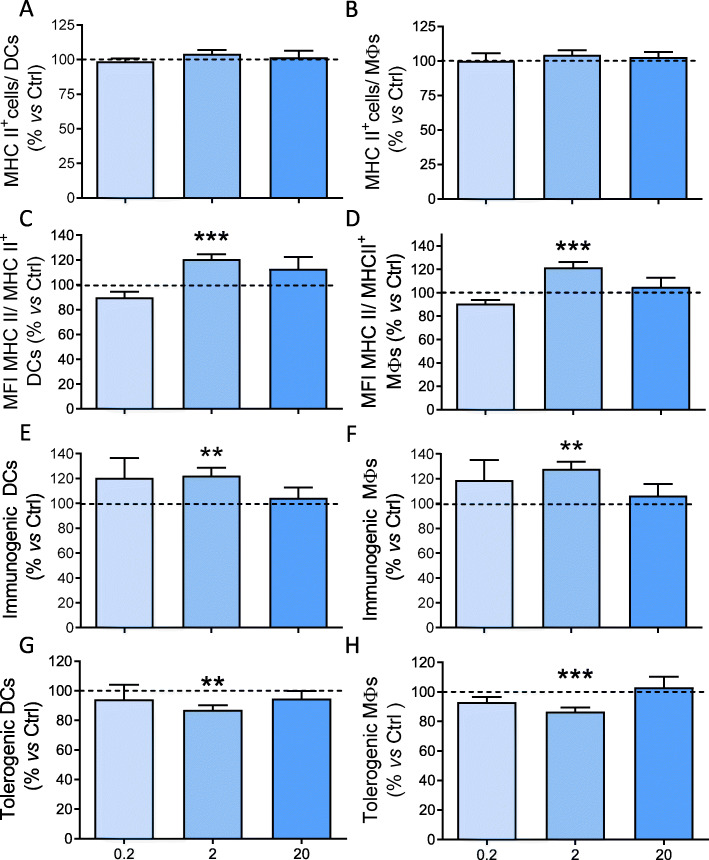
tPA polarizes splenic APCs from EAE mice towards a pro-inflammatory phenotype. Splenocytes extracted from EAE mice at the peak of the clinical course were treated in the indicated conditions. **a, b** Percentage of **a** CD11c^+^MHC-II^+^-DCs and **b** F4/80^+^ MHC-II^+^-Mɸ in each APC subpopulation. **c, d** MFI of MHC-II in APCs, **e–h** Percentage of **e, f** MHC-II^+^ CD80^+^ CD86^+^ immunogenic and **g, h** and MHC-II^+^ CD80^-^ CD86^-^ tolerogenic APCs in the presence of different tPA concentrations. In all cases, tPA at the concentrations of 0.2 and 20 μg/mL showed no differences. Results are expressed as mean + SEM (*N* = 5), ^*^P < 0.05

We then analyzed whether tPA may affect the proportion and the phenotype of DCs and Mɸ (Fig. 6, Suppl. Fig. 5). First, splenocytes extracted from EAE mice at the peak of the clinical course were treated with different concentrations of exogenous tPA (0.2, 2, and 20 μg/mL) during 24 h. None of the tPA concentrations modified the percentage of antigen presenting DCs (CD11c^+^MHC-II^+^) and Mɸ (F4/80^+^ MHC-II^+^) populations (Fig. 6a and b). However, tPA (2 μg/mL) induced a significant increase of MHC-II^+^ MFI in both cell populations compared to control conditions (DCs 120.75% ± 3.92; Mɸ 121.69% ± 4.72, *P* < 0.001, Fig. 6c and d). Splenic APCs modify their immunophenotype along the clinical course of EAE. The number of APCs with a more immunogenic profile (i.e., MHC-II^+^ CD80^+^ CD86^+^ CD40^+^ cells) increased along the clinical course of the disease, being maximum at the peak. In parallel, the number of APCs with a more tolerogenic immunophenotype (i.e., MHC-II^+^ CD80^-^ CD86^-^ PD-L1^+^ cells) decreased, being minimum at the peak and remained very high in asymptomatic EAE mice (suppl. Fig. 6). Interestingly, tPA (2 μg/mL) promoted the polarization on the MHC-II^+^ expressing antigen-presenting cell (APC) subsets towards a more pro-inflammatory and immunogenic phenotype, with a significant increase in the percentage of MHC-II^+^ CD80^+^ CD86^+^-APCs (DCs 122.45 ± 6.22, *P* = 0.002; Mɸ 128.04 ± 5.70; *P* = 0.004, Fig. 6e and f) and a decrease in the percentage of MHC-II^+^ CD80^-^ CD86^-^ tolerogenic APCs (DCs 87.09% ± 3.20, Mɸ 86.71% ± 2.70; *P* = 0.005 and *P* < 0.001, respectively; Fig. 6g and h).

**Fig. 7:**
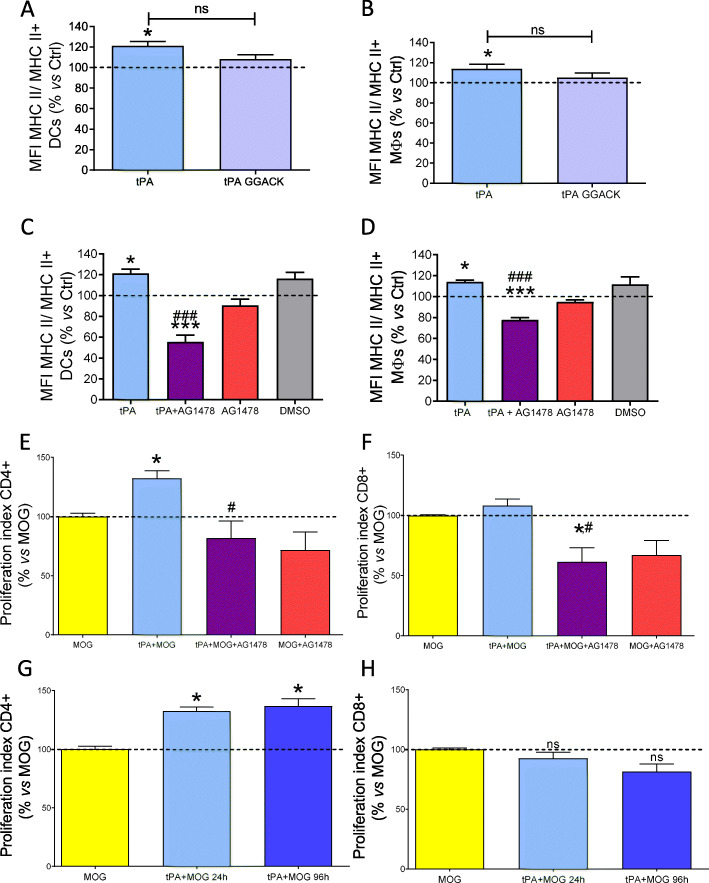
tPA stimulates MOG_35–55_-dependent APC maturation and T cell proliferation *via* its EGF-like domain. Splenocytes extracted from EAE mice at the peak of the clinical course were treated in the indicated conditions. **a, b** MHC-II MFI in **a** CD11c^+^MHC-II^+^-DCs and **b** F4/80^+^ MHC-II^+^-MΦs exposed to 2 μg/mL of tPA or 2 μg/mL of tPA-GGACK. **c, d** Expression of MHC-II MFI in **c** CD11c^+^MHC-II^+^-DCs and **d** F4/80^+^ MHC-II^+^-MΦs after combined treatment with 2 μg/mL of tPA and 5-μM EGFR inhibitor AG1478. **e, f** Cell proliferation index of **e** CD4^+^ and **f** CD8^+^ T cells after combined treatment with 5 μg/mL MOG_35–55_, 2 μg/mL of tPA and 5-μM EGFR inhibitor AG1478. *N* = 6–7 and ^*^P < 0.05, ^**^P < 0.01, ^***^P < 0.001 *vs* control; ^#^*P* < 0.05, ^###^*P* < 0.001 *vs* tPA. **g, h** Cell proliferation index of sorted **g** CD4^+^ and **h** CD8^+^ T cells in co-culture with MOG-stimulated CD3 T cell-depleted splenocytes with/without 2 μg/mL of tPA during 24 or 96 h (*N* = 6–7 for **a–f**, *N* = 3 for **g** and **h**, and ^*^P < 0.05, ^**^P < 0.01, ^***^P < 0.001 *vs* baseline; ^#^*P* < 0.05, ^###^*P* < 0.001 *vs* tPA)

Then, we aimed at analyzing the receptors and/or tPA functional domains enrolled in APC maturation. Treatment of APCs with catalytically inactive tPA (tPA-GGACK) did not modify MHC-II^+^ expression, although the difference with native tPA was not significant (tPA: DCs 121.12% ± 4.211, Mɸ 113.96% ± 4.406; *P* = 0.040 and *P* = 0,042, respectively, *versus* control condition; tPA GGACK: DCs 108.30% ± 4.166; Mɸ 105.21% ± 4.647, with respectively *P* = 0.156 and *P* = 0.440 *versus* control condition and *P* = 0.072; *P* = 0.186 versus tPA; Fig. 7a and b). Nonetheless, none of the tPA-mediated effects on APC polarization were modified by the addition of Glunomab® (Suppl. Fig. 7). These data indicate that the action of tPA on APCs is not mediated *via* interaction with NMDAR.

On the other hand, it has been shown that tPA can act via non-proteolytic “growth factor-like” effects [30], some of them mediated by its EGF-like domain [4, 15]. The addition of the EGFR blocking agent AG1478 decreased MHC-II^+^ expression on MHC-II^+^ APCs as compared with the condition with tPA (DCs 55.49% ± 6.525; Mɸ 77.76% ± 2.249; *P* < 0.001, Fig. 7c and d). These data indicate that tPA promotes APC maturation partly by the activation of EGFR.

### tPA-mediated APC polarization is accompanied by a higher MOG-specific T cell response

In order to determine whether the stimulatory effect of tPA on APC maturation may modify T cell functions, splenocytes extracted from EAE mice at the peak of the clinical course were cultured in the presence/absence of tPA, with or without *ex vivo* reactivation with MOG. In the absence of MOG reactivation, tPA by itself did not induce CD4^+^ or CD8^+^ T cell proliferation (CD4^+^ 107.22% ± 10.94; CD8^+^ 124.73% ± 57.01 data not shown). Condition with MOG reactivation showed a higher percentage of proliferation than control conditions for both CD4^+^ and CD8^+^ T cells (CD4^+^ 852.79% ± 125.03; CD8^+^ 1525.90% ± 261.96, both *P* < 0.05, data not shown). Interestingly, tPA potentiated MOG-induced CD4^+^ T cell proliferation (132.43% ± 6.28; *P* < 0.05, Fig. 7e). In line with the previous results about APC maturation, the stimulatory effect of tPA on MOG-activated CD4^+^T cell proliferation was abrogated when EGFR activity was blocked by AG1478 (81.88% ± 14.25; *P* < 0.05, Fig. 7e), while AG1478 did not modify MOG-induced APC proliferation in the absence of tPA (Fig. 7e). Remarkably, the stimulatory effect of tPA was not observed in MOG-activated CD8^+^ T cells (108.26% ± 5.45; *P* > 0.05, Fig. 7f), in accordance with what was observed in the context of CD3/CD28 polyclonal activation of CD8^+^ T cells extracted from tPA^-/-^ mice (Fig. 3c).

Finally, to assess whether tPA can act directly on APCs, CD3 T cell-depleted splenocytes isolated from EAE mice at the peak of the clinical course were stimulated by MOG peptide in the presence/absence of tPA. After 24 h, Tag-it Violet-labelled sorted CD3 T cells from the spleen of EAE mice at the peak of the clinical course were added, and the medium was replenished in all culture conditions. In one experimental condition, tPA was removed at 24 h to check the specific effect of tPA over CD3 T cell-depleted splenocytes. Our data showed that tPA pretreatment of CD3 T cell-depleted splenocytes during the first 24 h increased their ability to potentiate CD4 T cell proliferation (MOG + tPA 24 h: 132.47% ± 3.52; *P* < 0.05 versus MOG, Fig. 7g). Interestingly, the presence of tPA during the whole experiment did not induce an additional proliferative effect on CD4 T cells (MOG + tPA 96 h: 136.81% ± 6.22; *P* < 0.05 versus MOG, Fig. 7g). On the other hand, tPA did not exert any significant effect on CD8 T cells (MOG + tPA 24 h: 92.55% ± 5.14; MOG + tPA 96 h: 81.55% ± 6.25, Fig. 7h). These results indicate that tPA can act directly on APC maturation during the first 24 h of exposition to potentiate MOG-activated CD4 T cell proliferation.

In sum, our data indicated that the direct and early effect of tPA on APC maturation and pro-inflammatory polarization resulted in amplified MOG-induced CD4^+^ T cell response. This effect may explain the deleterious role of tPA in EAE.

## Discussion

Previous works reported that tPA expression is increased in the CNS during EAE [11, 31], a result which suggests an involvement of this serine protease in the EAE physiopathology. Although tPA has been reported to induce neurotoxic effects [32], these effects are unlikely to play a significant part in the processes reported here. In fact, genetically induced upregulation of tPA expression in neurons does not impact the score of EAE [31]. Furthermore, the motor symptoms observed in EAE correlate with both demyelination [33] and axonal injury [34] but not with neuronal death, which is hardly detectable in the spinal cord of MOG-induced EAE until late stages [35]. Thus, the pathophysiology of EAE and the timing of the effects observed in this work, where differences between tPA^-/-^ and WT mice occur at early symptomatic stages, also argue against a significant implication of neuronal death.

Indeed, our study reports a deleterious role of tPA in EAE by an action on lymphoid and myeloid cell subsets by two complementary mechanisms: on the one hand, tPA directly increases T cell activation, proliferation and IL-6 secretion in a plasmin-dependent manner. On the other hand, tPA indirectly increases T cell proliferation by increasing the expression of MHC-II and co-stimulatory molecules in DCs and Mɸ, by both proteolytic and growth factor-like effects. These elements may bring an explanation to the deleterious effect of endogenous tPA observed here in EAE and put forward complementary mechanisms of tPA in immune response.

Previous data from the literature are contradictory about the role of tPA in neuroinflammation in EAE. Some reports indicate that tPA^-/-^ mice present earlier and more severe disease than their WT counterparts, suggesting a protective role of tPA [12, 13, 36]. Other studies using tPA^-/-^ mice, including the present one, argue for a deleterious effect of tPA [11]. These discrepancies could be explained by different experimental conditions, including different MOG doses, the use of MOG re-boost or differences in the age of mice, but also in environmental conditions [37]. These different conditions determine different EAE severity profiles between laboratories, in which the beneficial and deleterious effects of tPA [38] may then express themselves differently. Nevertheless, repeated administration of tPA worsened the clinical scores of EAE and slightly anticipated its onset [39], which supports an adverse effect of tPA in this model. Moreover, our data showing that tPA enhances IFN-γ, a cytokine with encephalitogenic properties, are also in favor of a deleterious role of tPA in EAE.

The fact that tPA promotes T cell activation and proliferation is in agreement with recently published data [36]. The present study provides further elements by demonstrating that this effect is dependent of plasminogen activation into plasmin. However, the potential mechanisms downstream of plasmin activation that may act on T cells in the context of EAE remain elusive. At least three potential candidates can be cited. First, tPA induces the release of the pro-form of matrix metalloproteinase 9 (pro-MMP9) from endothelial cells by activating the lipoprotein receptor-related protein-1 (LRP) receptor [40] and activates pro-MMP9 into its active form, MMP9 *via* plasminogen activation [41]. Remarkably, MMP9 is involved in T cell proliferation, as MMP9-deficient T cells display altered proliferative ability [42]. Second, tPA activates heparin-bound EGF into free EGF, *via* the action of plasmin and matrix MMP9, leading to EGFR signalling in different cell types [43]. Third, plasmin is able to raise the bioavailability of the chemokine CCL21, known to regulate the homing of T cells and DCs towards lymphoid organs [44] and to act as a co-stimulatory molecule to promote T cell expansion and Th1 differentiation [45]. Interestingly, EGFR inhibition induces CD4^+^ T cell anergy *in vitro* and *in vivo* [46]. Further experiments may clarify whether MMP9, EGFR, and/or CCL21 participate in the mechanisms by which tPA promotes T cell proliferation and activation.

Our data emphasize plasmin-dependent immune functions of tPA in EAE. In accordance with our results, plasminogen deficiency in mice delays the onset and protects from demyelination in EAE [47]. Furthermore, plasmin is able to activate microglia [48], suggesting that tPA/plasmin axis is an important component to consider in future studies on the biological mechanisms underlying EAE.

Our data show that tPA acts also on myeloid cells by a distinct mechanism involving EGFR activation. By this action, tPA increases the expression of MHC-II and the co-stimulatory molecules CD80 and CD86 by DCs and Mɸ, leading to a polarization from a tolerogenic to an immunogenic activity state. Our data did not allow to firmly conclude on a possible implication of the proteolytic activity of tPA in these effects. This possibility should be more thoroughly addressed in further studies.

Our results were in contrast with a previous study reporting that tPA reduces macrophage response to LPS by inhibiting ERK pathway and cytokine production, an effect mediated *via* the combined action of NMDAR and LRP pathways [20]. The mechanisms implied are likely to be different from what described in the present work: the increase in antigen-presenting capacity of APCs, induced by tPA, is not prevented by Glunomab®, which points to the involvement of another pathway. In fact, in our co-culture system, the CD4 T cell proliferative effect of tPA on APC is reversed by EGFR inhibition, which indicates that tPA-mediated effects on antigen presentation are exerted *via* EGFR activation. The direct EGFR-mediated effect on T cell proliferation cannot be totally discarded in light of our observations. Nevertheless, pre-treatment of T cell-depleted splenocytes with tPA is sufficient to potentiate the proliferation of T cells added subsequently to the co-culture (Fig. 7g and h). This indicates that tPA-mediated effects on antigen presentation are directly exerted via EGFR and essentially mediated via an action on APCs. Our study is the first that correlates the expression of MHC-II molecules with the activation of EGFR by tPA. However, the downregulation of MHC-II expression by AG1478 in the absence of tPA may indicate that EGFR is intrinsically involved in antigen presentation. Interestingly, EGFR downstream signalling inhibition is a promising strategy in diverse tumor types by inducing MHC-II in APCs and breaking down tolerance [49]. Our data reinforces the idea of targeting EGFR activity as an interesting target to modulate MHC-II expression, either to potentiate it, as in the case of cancer, or to reduce it, as in the case of MS or other autoimmune diseases in which tolerance induction is one of the golden aims in cell therapy-based strategies [50].

These data show that tPA can act on the myeloid population by mechanisms independent of plasmin generation and complete previous reports showing that tPA can activate microglial cells *via* its finger domain through annexin II [30]. In order to gain insights into the primary target cells of tPA action in EAE, further studies may be conceived. For instance, passive EAE experiments may be induced with encephalitogenic T cells that would be treated with tPA prior to adoptive transfer.

tPA-induced enhancement of IL-6 cytokine production by activated splenocytes is also in favor of a pro-inflammatory role of tPA in EAE. Indeed, this cytokine is crucial for neuroinflammation as illustrated by the fact that IL-6-deficient mice are resistant to EAE [51]. However, tPA also intriguingly increases IL-10 production, a cytokine with immunomodulatory functions. However, it should be kept in mind that IL-10, as many cytokines, may have dual effects depending on the context. For example, IL-10 may have immunostimulating properties by promoting the generation of cytotoxic T cells [52] and NK cells [53], or favoring the germinal center response [54]. So, further works are needed to explore the net effect of tPA on the cytokine network and neuroinflammation in the course of EAE.

## Conclusion

Our study opens new clues into the mechanisms by which tPA/plasmin axis participates in the pathogenesis of MS and its animal models. These mechanisms could be involved also in other neurological diseases such as Alzheimer’s disease, amyotrophic lateral sclerosis and Parkinson’s disease in which T cells are also involved [55]. Our study paves the way for further studies investigating the downstream targets involved in the proinflammatory effects of tPA in T cells and myeloid cells.

## Supplementary Information

**Additional file 1: Suppl Figure 1: Gating strategy and lymphoid cell response after EAE in WT and tPA**^**-/-**^ **mice. (A)** Representative gating strategy for absolute cell count analysis of leukocyte subsets in spinal cord homogenates (WT mouse, plateau phase of EAE)**. (B)** CD3+ leukocytes were gated for subpopulation analysis after using scattergram gates for viable leukocytes (in A). (C) representative flow cytometry plots for the assessment of intracellular cytokines IFNγ and IL-17 in CD4+ cells after EAE in WT and tPA^-/-^. **Suppl Figure 2: T cell infiltration in the spinal cord of WT and tPA**^**-/-**^ **EAE mice.** Photomicrographs show representative images (from N=3) of indicated markers in spinal cord tissue samples of WT and tPA^-/-^ mice at the EAE plateau phase (d20±3) from **(A)** cervical, **(B)** upper thoracic, and **(C)** lumbar sacral. **Suppl Figure 3: Glunomab does not prevent the effect of tPA on T cells.** Splenocytes activated with both anti-CD3ε and anti-CD28 antibodies were treated or not with tPA at 10 μg/mL in presence of Glunomab or the isotypic antibody for 4 days. Proliferation index (%) of **(A)** CD4^+^ and **(B)** CD8^+^ T cells in the indicated conditions. Results are expressed as mean + SEM (N=3). *P<0.05 *vs* control #P< 0.05 *vs* indicated experimental group. **Suppl Figure 4: Myeloid response of WT and tPA**^**-/-**^ **mice at the plateau phase of EAE.** Representative gating strategy for absolute cell count analysis of leukocyte subsets in spinal cord homogenates of WT and tPA^-/-^ mice. CD3^-^ leukocytes were gated for subpopulation analysis after using scattergram gates for viable leukocytes (see Suppl.fig 1A)**. Suppl Figure 5: Myeloid response to tPA treatment.** Representative flow cytometry plots for **(A)** dendritic cells and **(B)** macrophages after tPA treatment (0-20 μg/mL). **Suppl Figure 6: Dynamic modification of immunogenic and tolerogenic DC phenotype along the EAE clinical course.** CD40 is highly present at the moment of maximum affectation and decreased when symptoms partially recover. PD-L1 is increased after the recovery of the clinical symptoms. Asymptomatic immunized mice showed a high presence of PD-L1^+^ tolerogenic DCs. C.S= clinical score and d.p.i= day post-immunization. **Suppl Figure 7: Effect of tPA on APC maturation is not mediated by its ability to interact with NMDA receptor.** Splenocytes extracted from EAE mice at the peak of the clinical course were treated with 2 μg/mL tPA in presence of Glunomab or an isotypic antibody (A-H). The percentages of MHC-II^+^CD80^+^CD86^+^ immunogenic and MHC-II^+^CD80^-^CD86^-^ tolerogenic APCs were determined by flow cytometry. Results are expressed as mean + SEM (N=5); *P< 0.05, **P<0.01 and ***P<0.001.

**Additional file 2: Suppl. Table 1**: Antibodies used in the present study. Each antibody is indicated with its target antigen, host species, clone number, isotype and commercial reference

## Data Availability

The data generated during this study are available from the corresponding author on reasonable request.

## Abbreviations

BBB: Blood—brain barrier
CSF: Cerebrospinal fluid
DC: Dendritic cells
dpi: Day post-immunization
EGF: Epidermal growth factor
EAE: Experimental autoimmune encephalomyelitis
IFN-γ: Interferon-γ
IL: Interleukin
Mɸ: Macrophages
MHC: Major histocompatibility complex
MOG: Myelin oligodendrocyte glycoprotein
MMP: Matrix metalloproteinase
MS: Multiple sclerosis
NMDAR: N-methyl-D-aspartate receptors
tPA: Tissue-type plasminogen activator
