# Supplementary material for: Tissue plasminogen activator worsens experimental autoimmune encephalomyelitis by complementary actions on lymphoid and myeloid cell responses
Source: J Neuroinflammation. 2021 Feb 20;18:52. doi: 10.1186/s12974-021-02102-5 (PMC7897384; doi:10.1186/s12974-021-02102-5)

Figure 1

Hélie et al., Tissue plasminogen activator worsens experimental autoimmune encephalomyelitis by complementary actions on lymphoid and myeloid cell responses

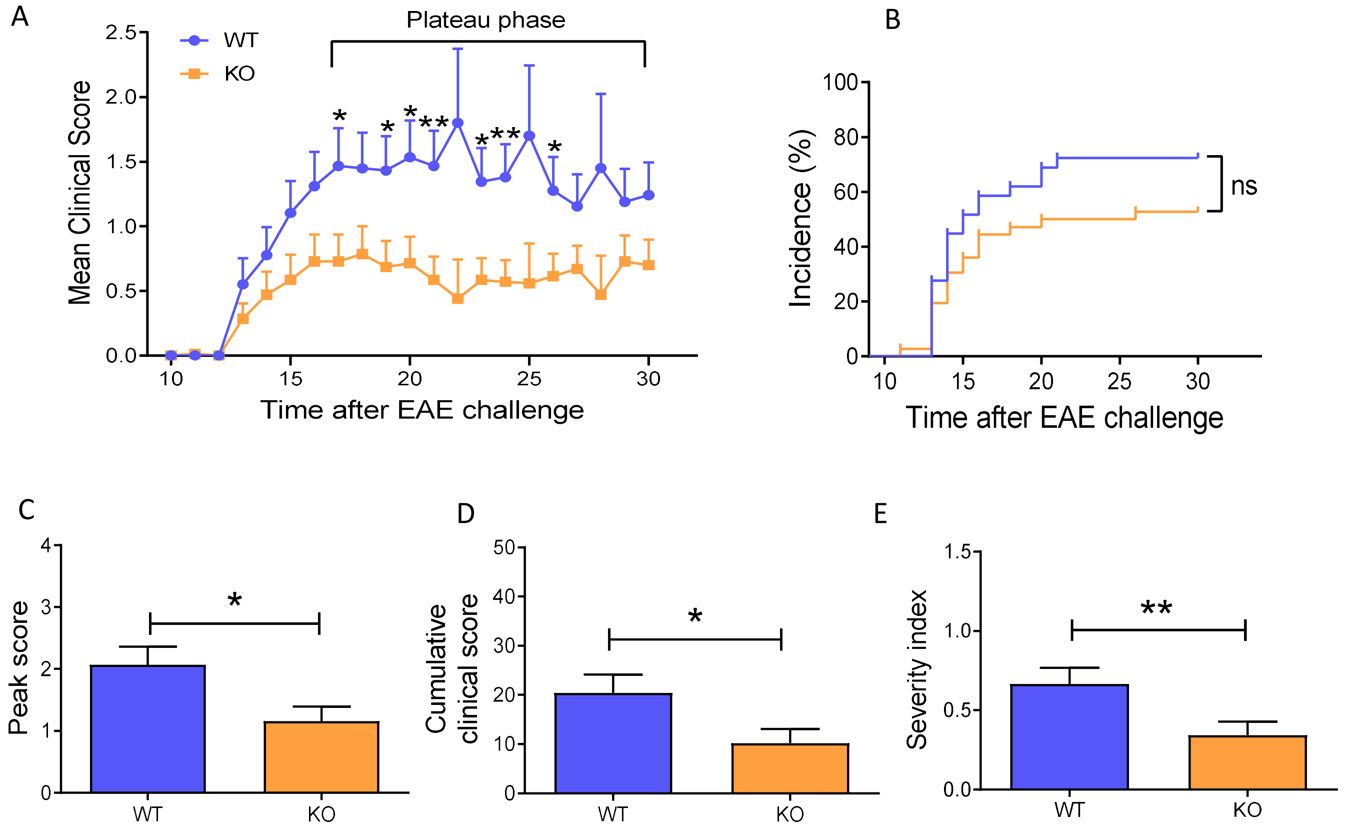

Figure 2

Hélie et al., Tissue plasminogen activator worsens experimental autoimmune encephalomyelitis by complementary actions on lymphoid and myeloid cell responses

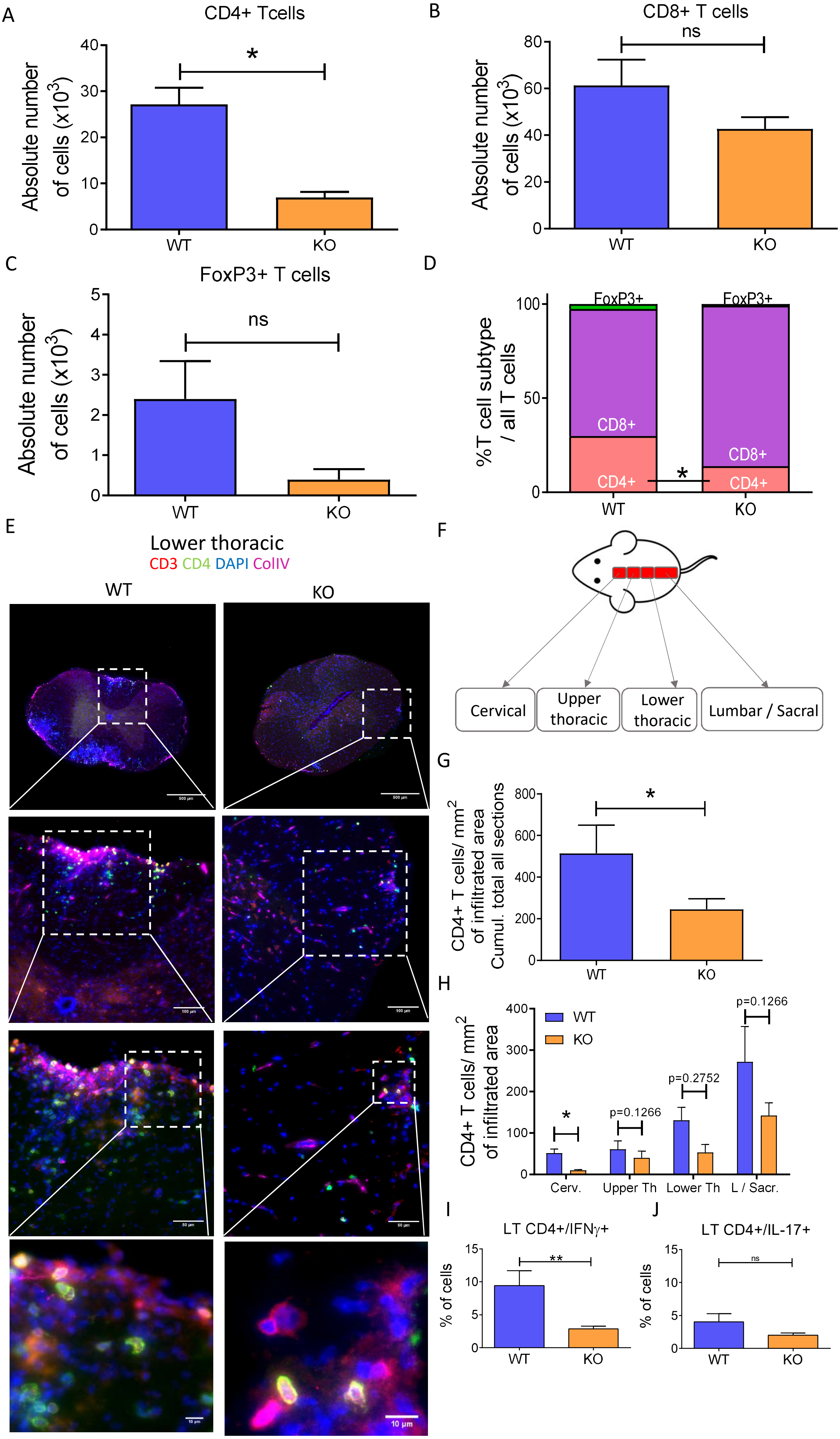

Figure 3

Hélie et al., Tissue plasminogen activator worsens experimental autoimmune encephalomyelitis by complementary actions on lymphoid and myeloid cell responses

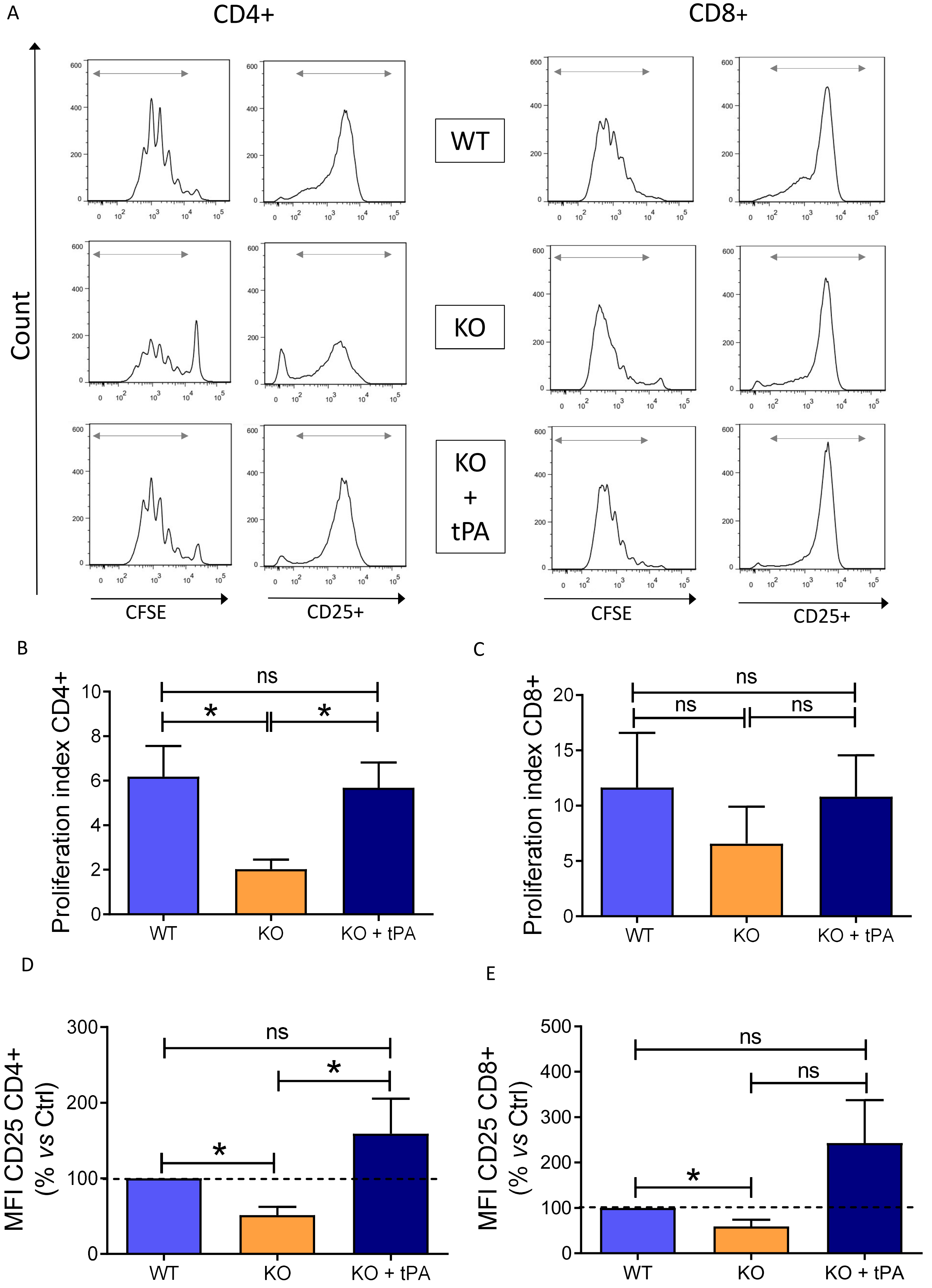

Figure 4

Hélie et al., Tissue plasminogen activator worsens experimental autoimmune encephalomyelitis by complementary actions on lymphoid and myeloid cell responses

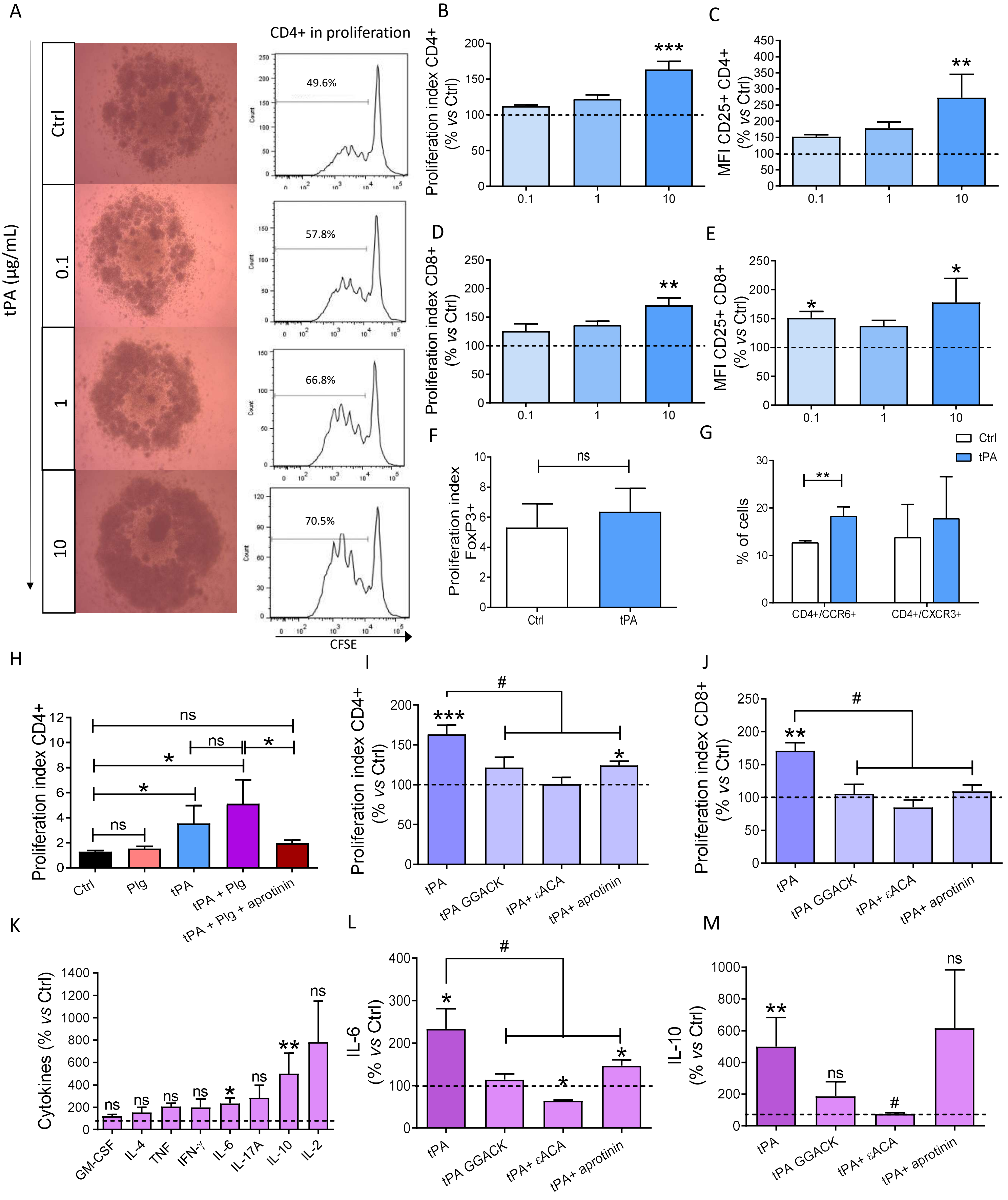

Figure 5

Hélie et al., Tissue plasminogen activator worsens experimental autoimmune encephalomyelitis by complementary actions on lymphoid and myeloid cell responses

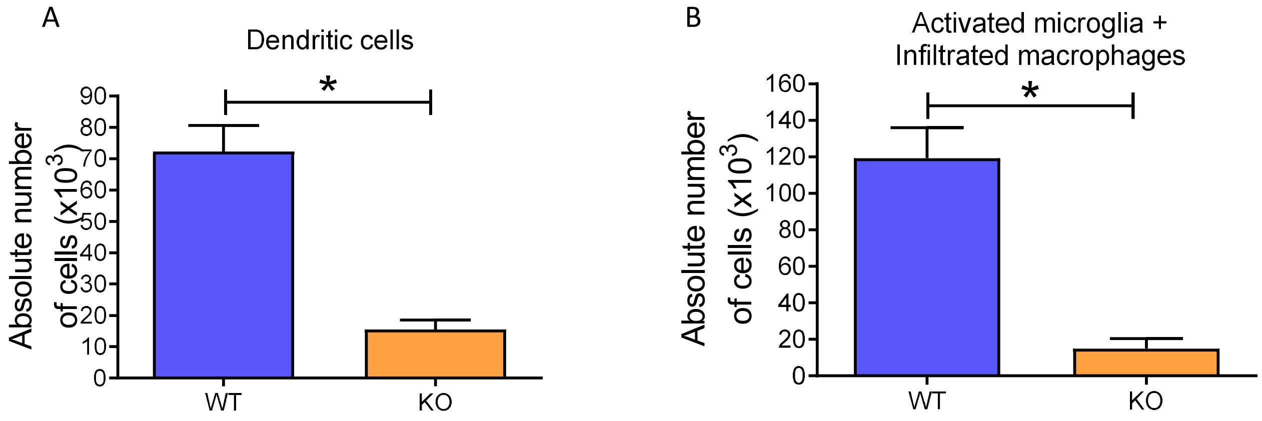

Figure 6

Hélie et al., Tissue plasminogen activator worsens experimental autoimmune encephalomyelitis by complementary actions on lymphoid and myeloid cell responses

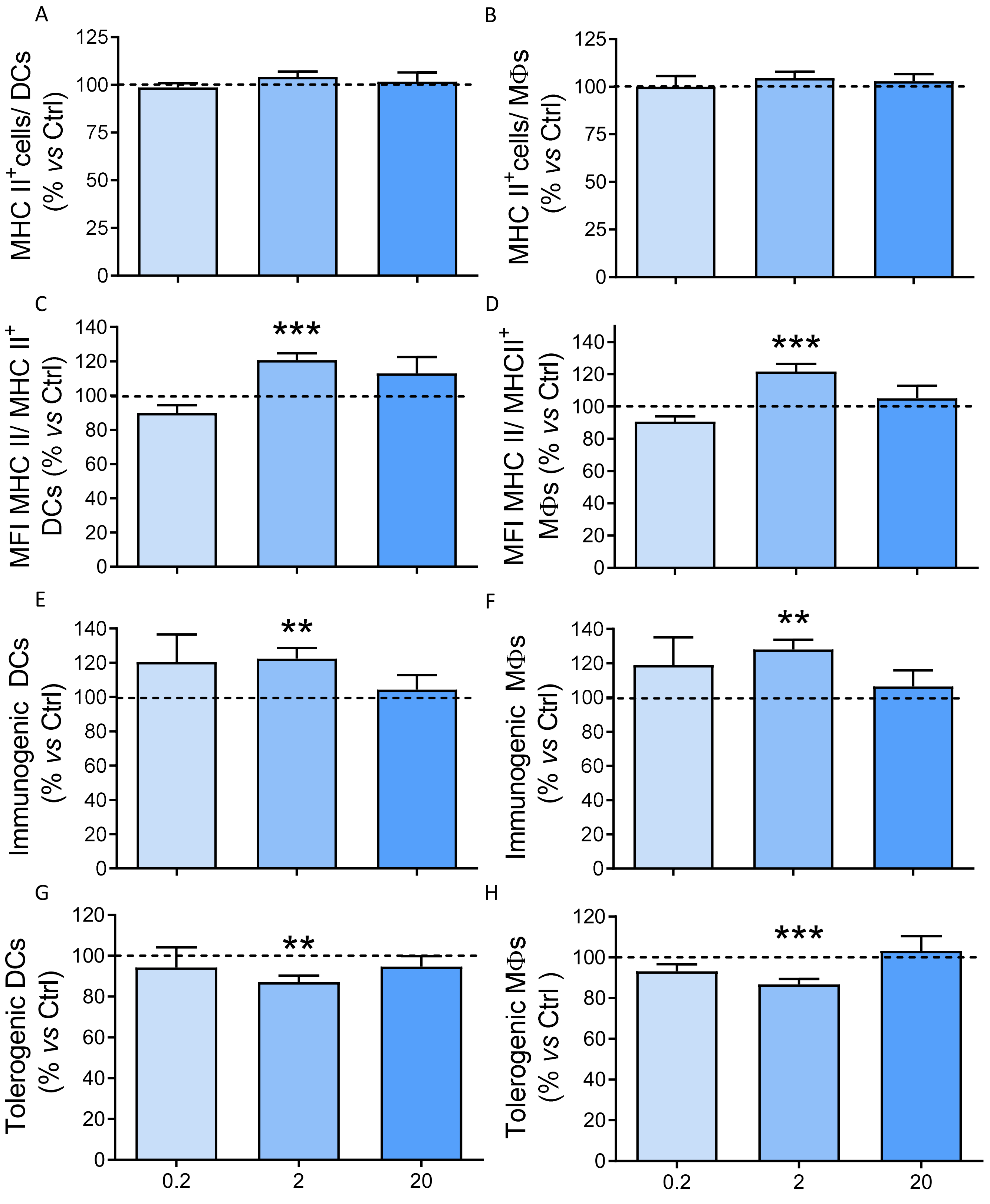

Figure 7

Hélie et al., Tissue plasminogen activator worsens experimental autoimmune encephalomyelitis by complementary actions on lymphoid and myeloid cell responses

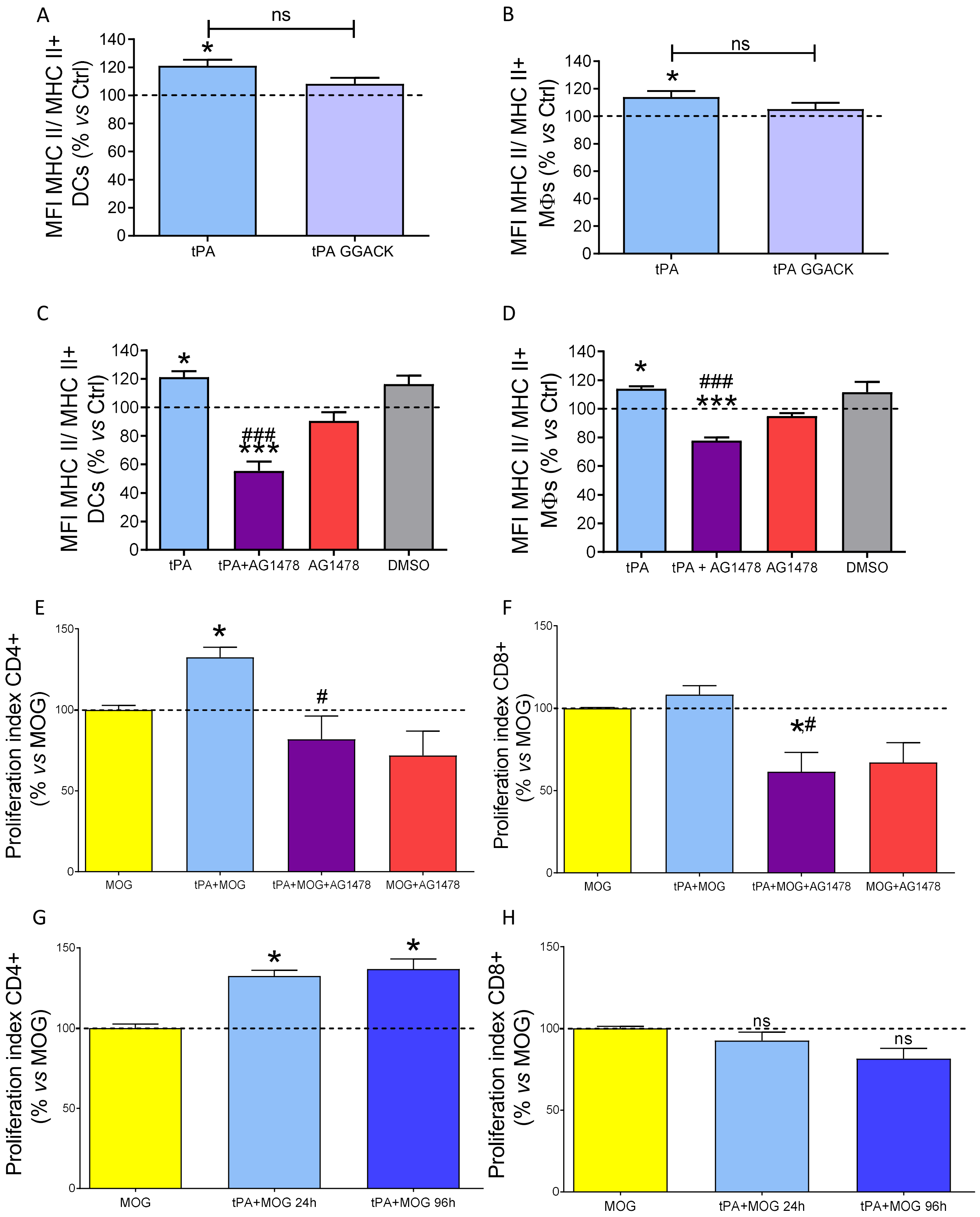

Suppl. 1

Hélie et al., Tissue plasminogen activator worsens experimental autoimmune encephalomyelitis by complementary actions on lymphoid and myeloid cell responses

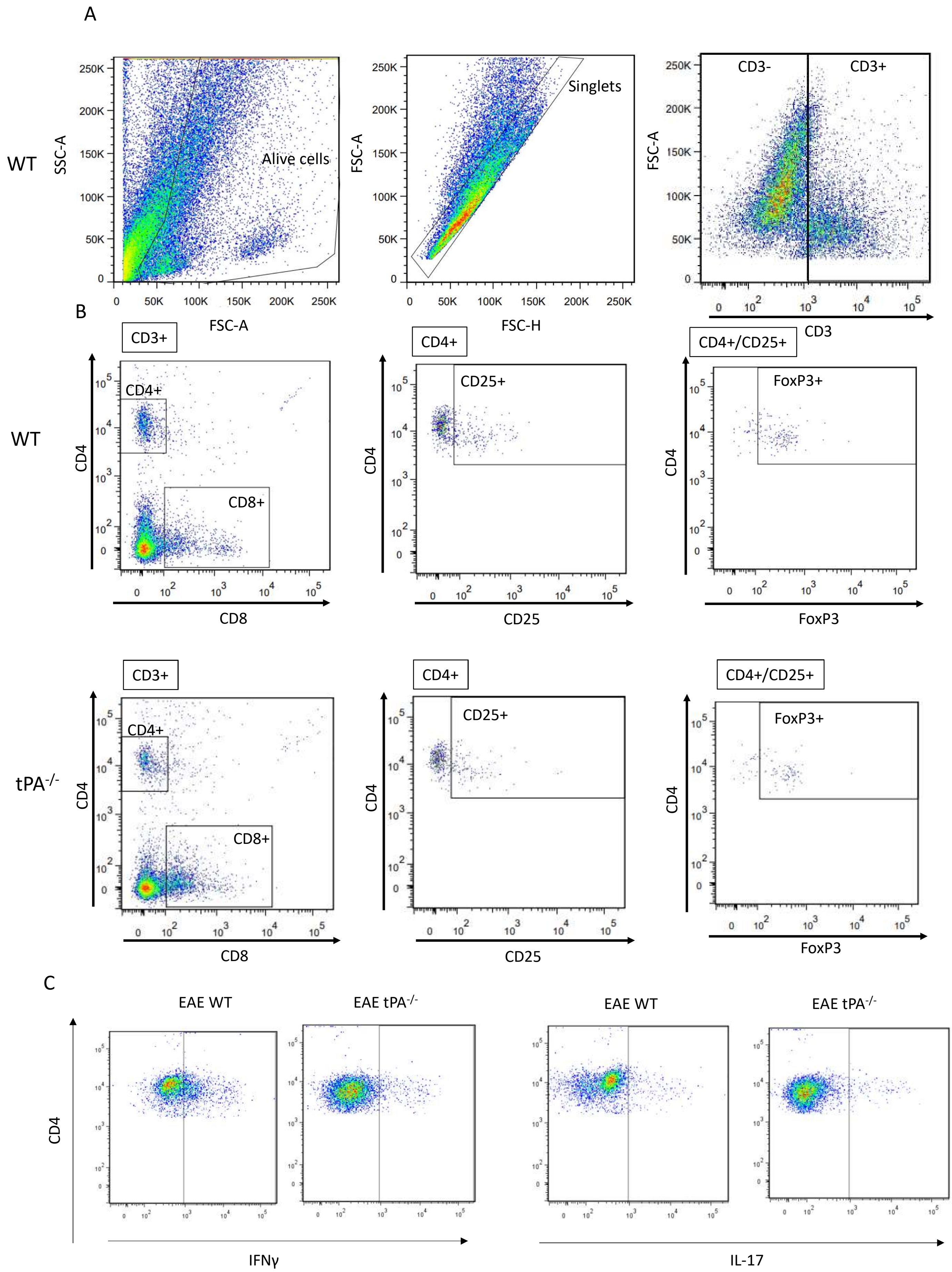

Suppl. 2

Hélie et al., Tissue plasminogen activator worsens experimental autoimmune encephalomyelitis by complementary actions on lymphoid and myeloid cell responses

A

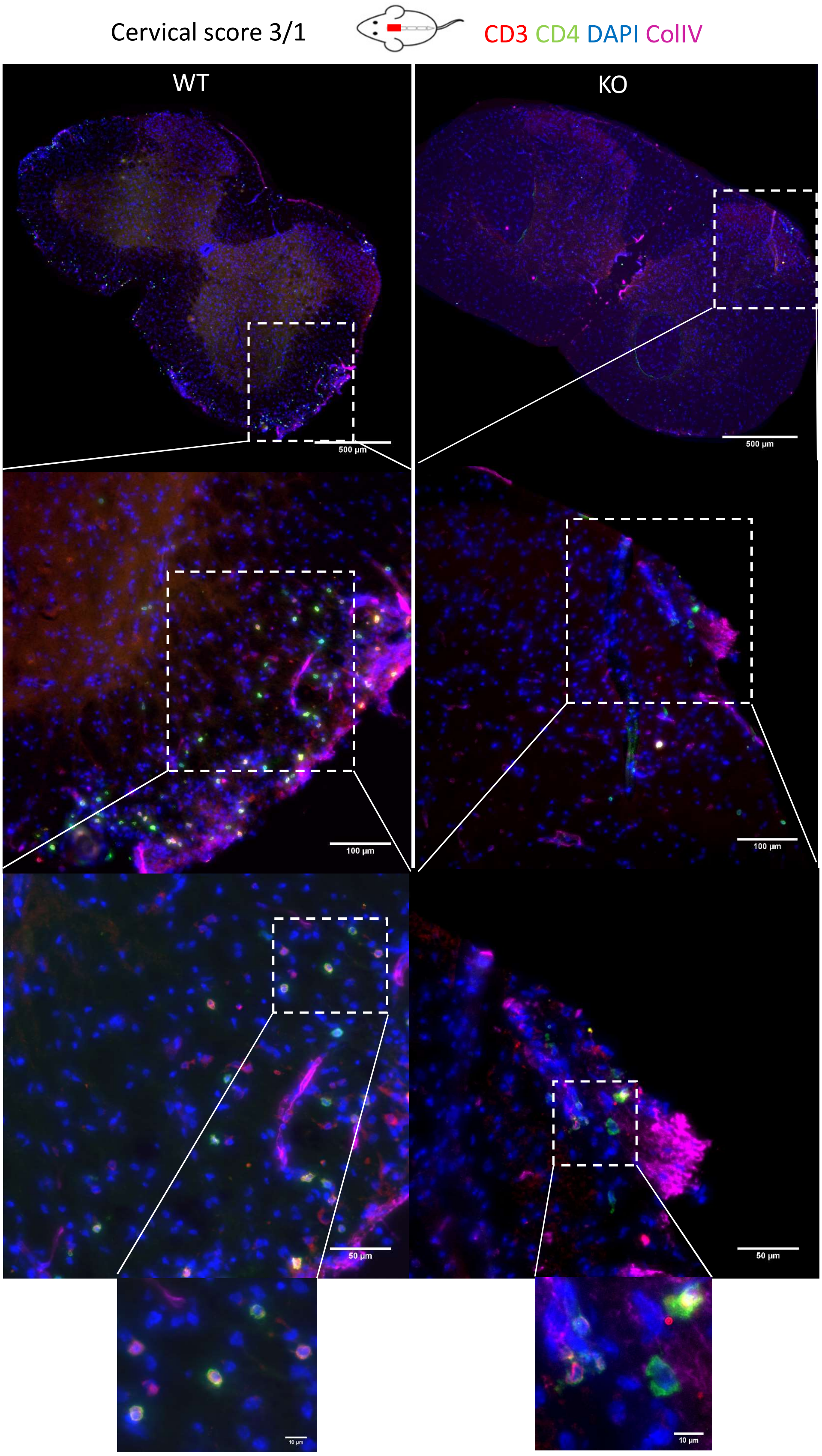

Suppl. 2

Hélie et al., Tissue plasminogen activator worsens experimental autoimmune encephalomyelitis by complementary actions on lymphoid and myeloid cell responses

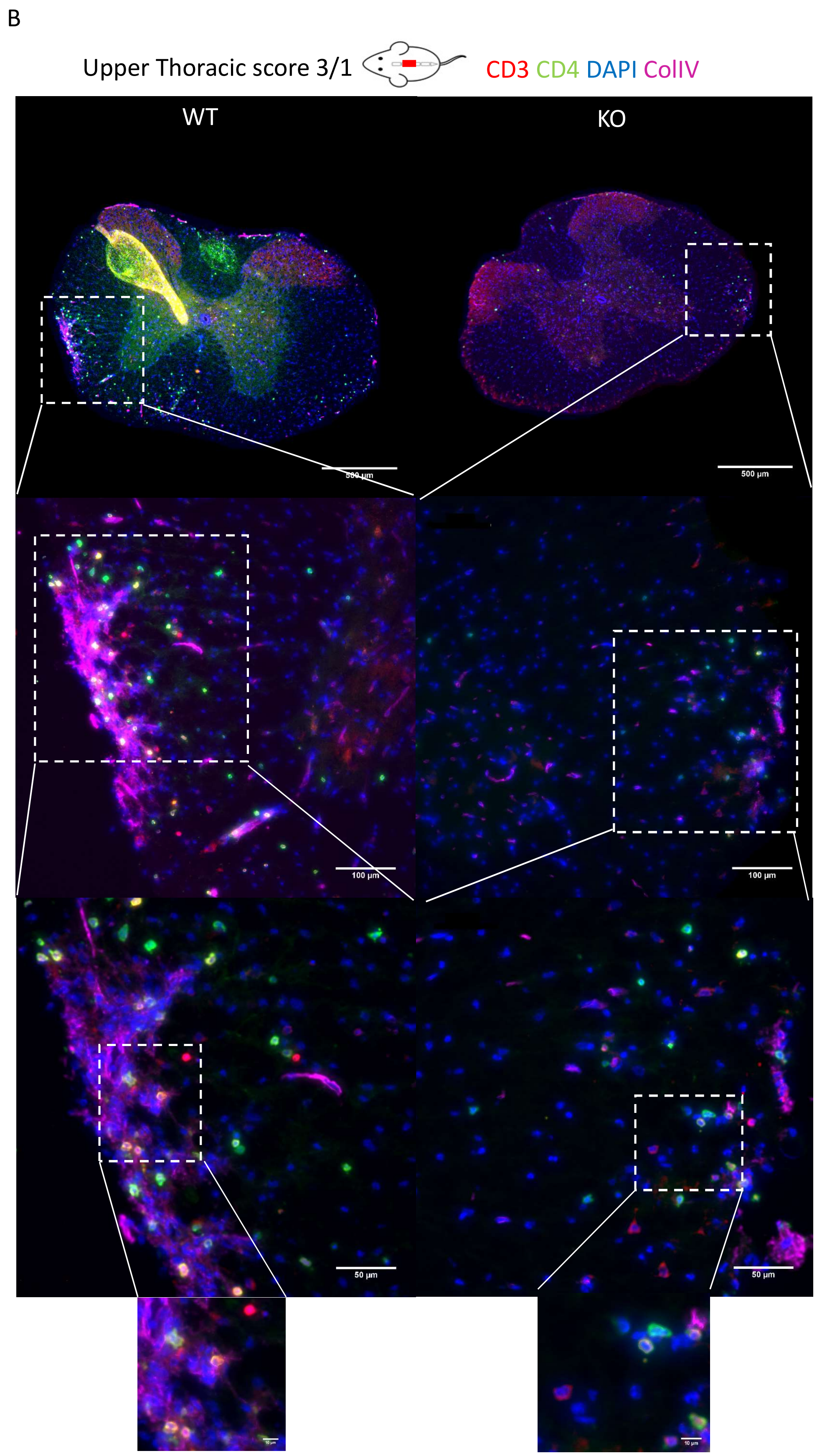

Suppl. 2

Hélie et al., Tissue plasminogen activator worsens experimental autoimmune encephalomyelitis by complementary actions on lymphoid and myeloid cell responses

C

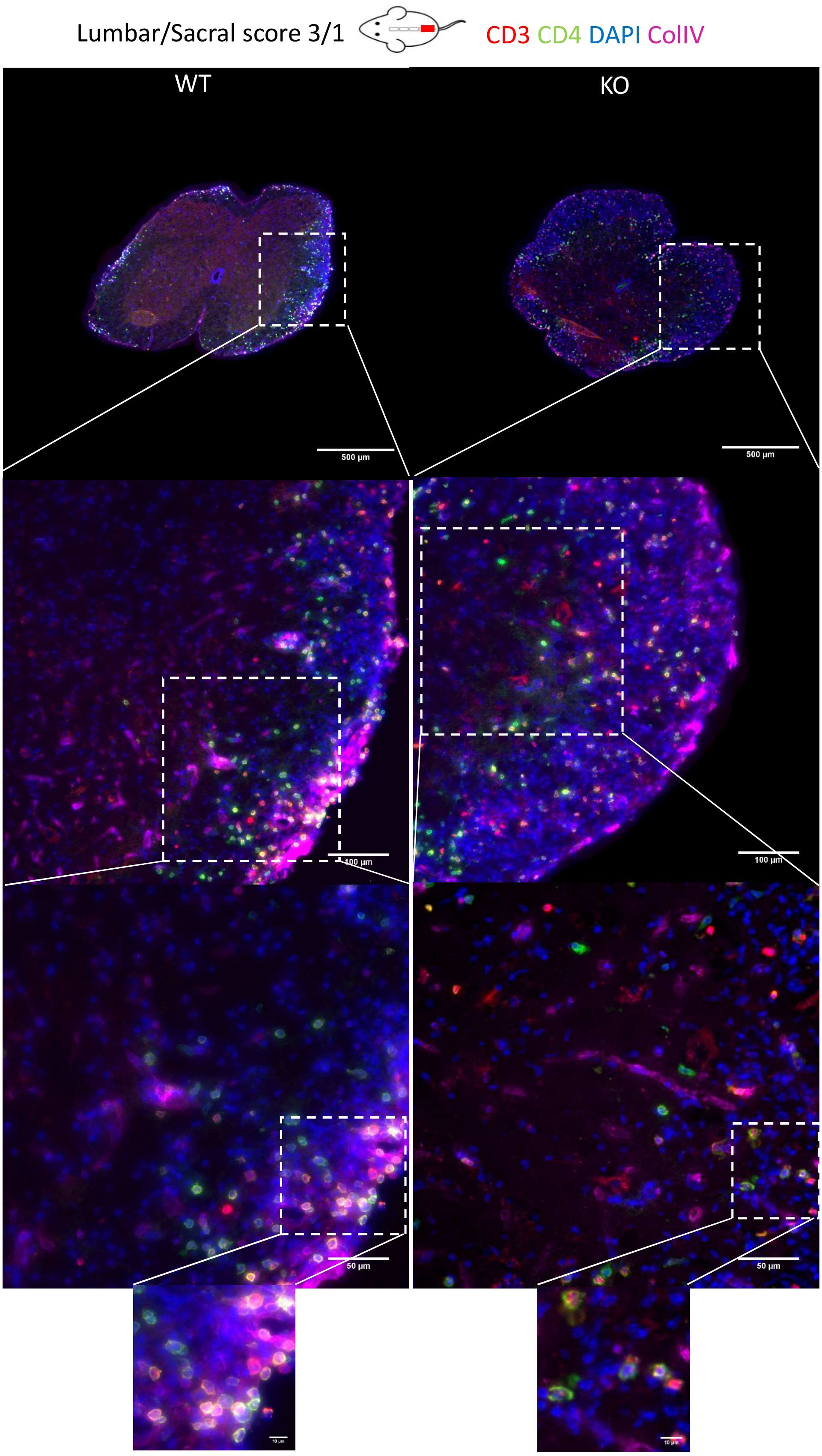

Suppl. 3

Hélie et al., Tissue plasminogen activator worsens experimental autoimmune encephalomyelitis by complementary actions on lymphoid and myeloid cell responses

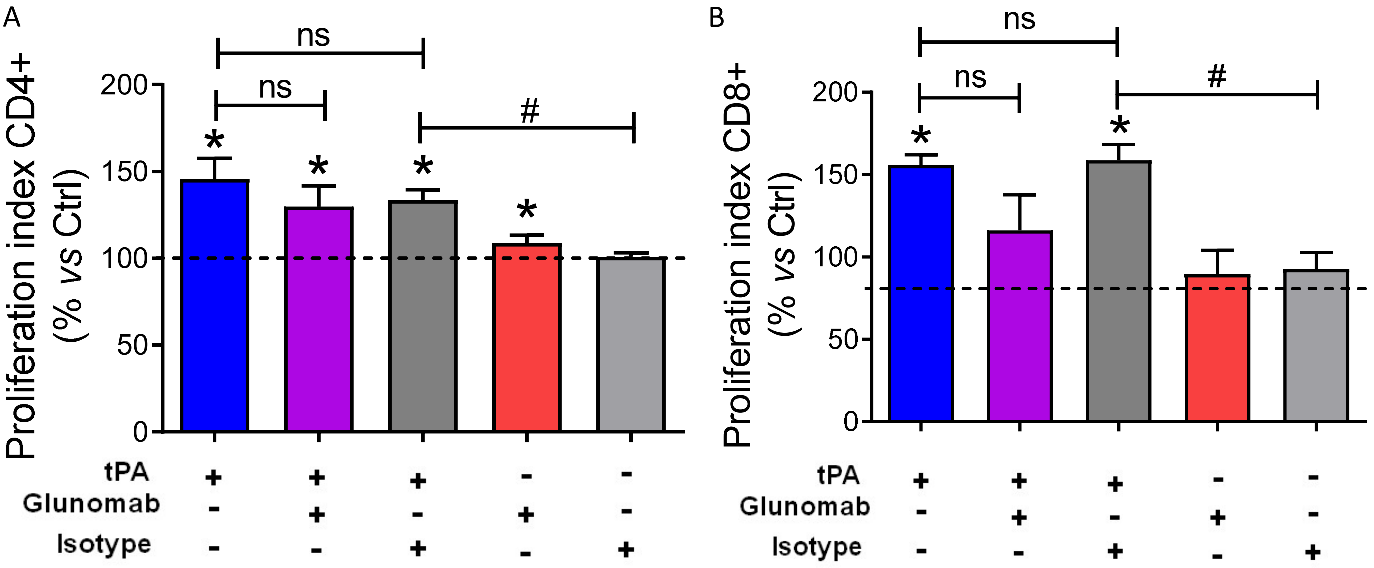

Suppl. 4

Hélie et al., Tissue plasminogen activator worsens experimental autoimmune encephalomyelitis by complementary actions on lymphoid and myeloid cell responses

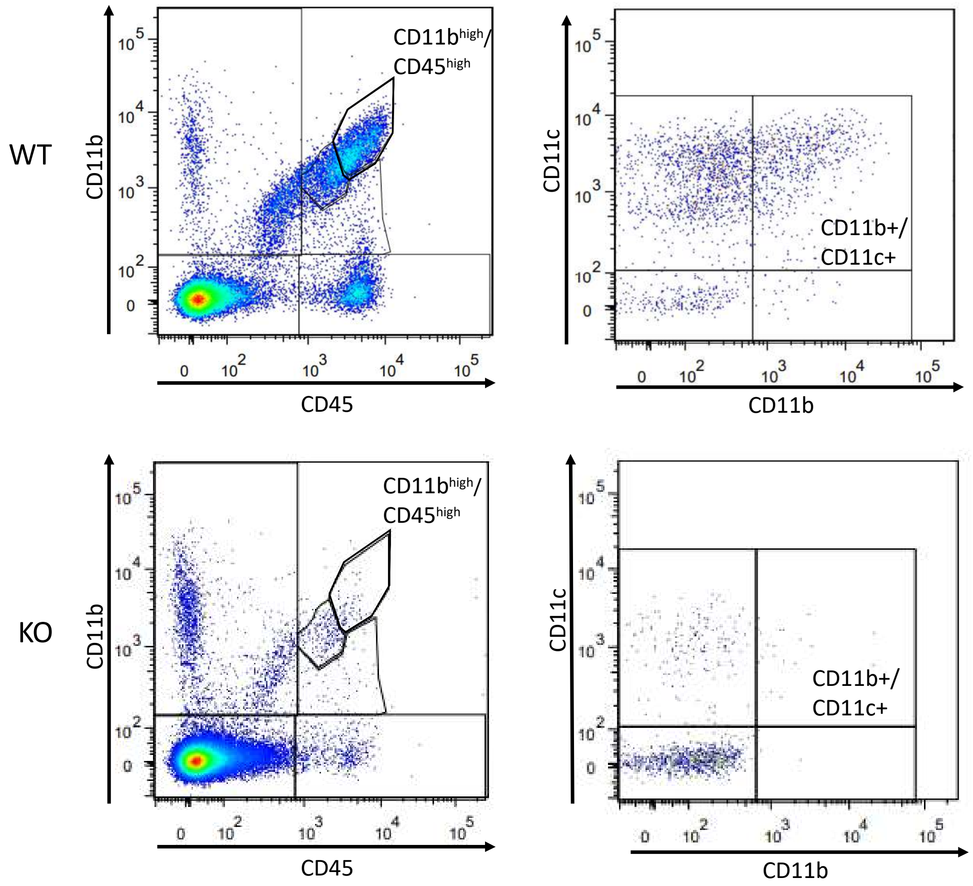

Suppl. 5

Hélie et al., Tissue plasminogen activator worsens experimental autoimmune encephalomyelitis by complementary actions on lymphoid and myeloid cell responses

A

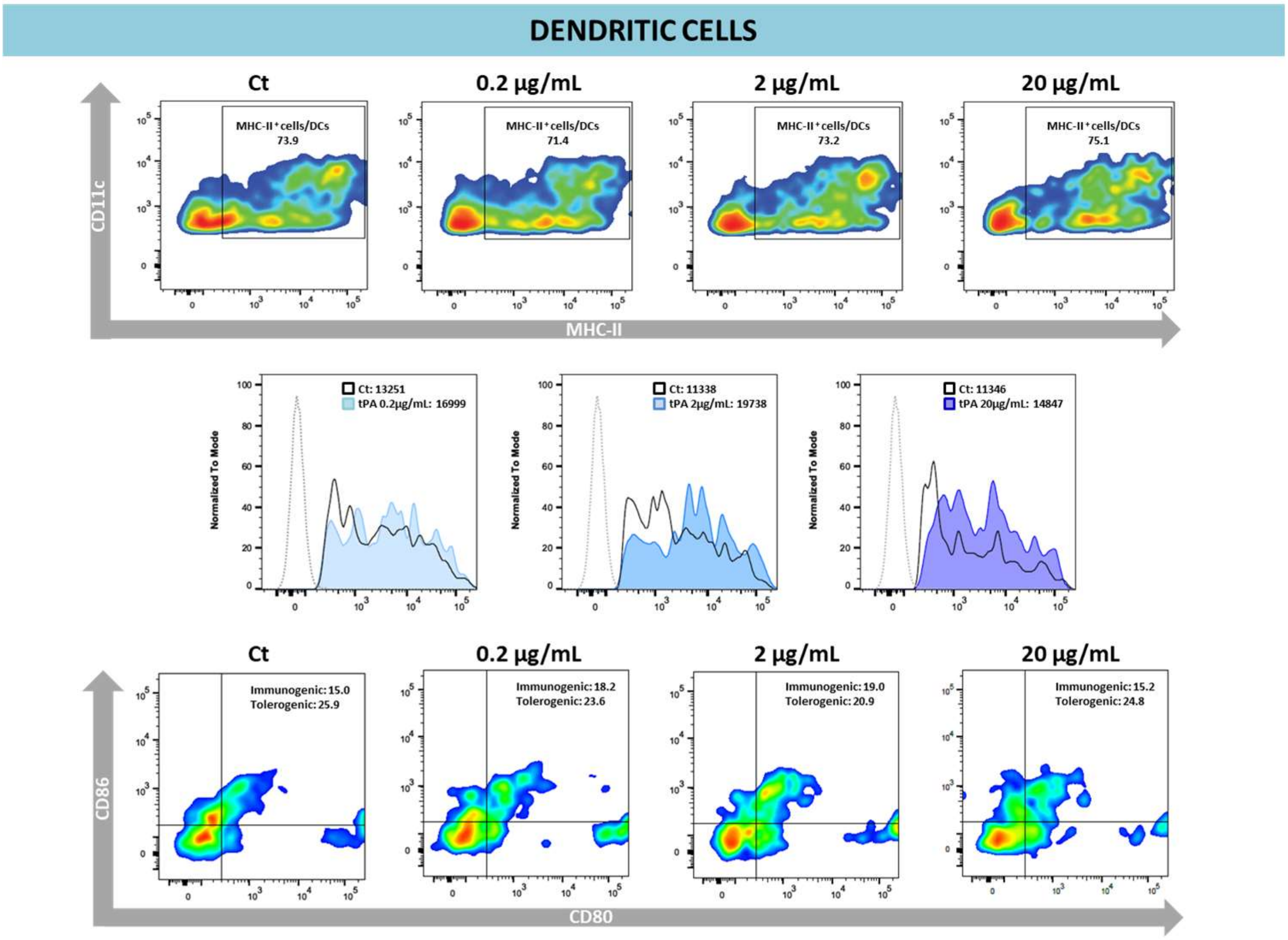

B

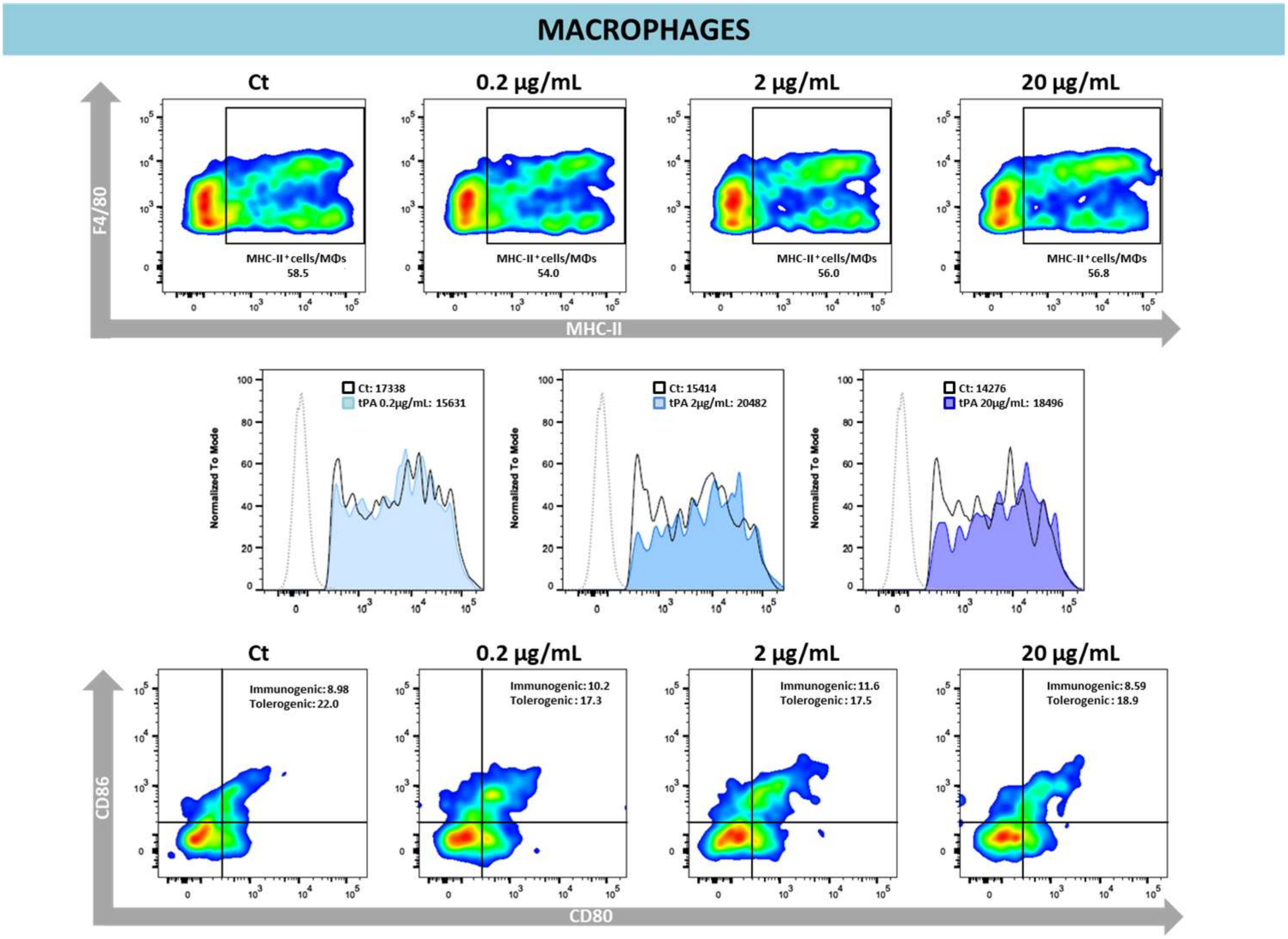

Suppl. 6

Hélie et al., Tissue plasminogen activator worsens experimental autoimmune encephalomyelitis by complementary actions on lymphoid and myeloid cell responses

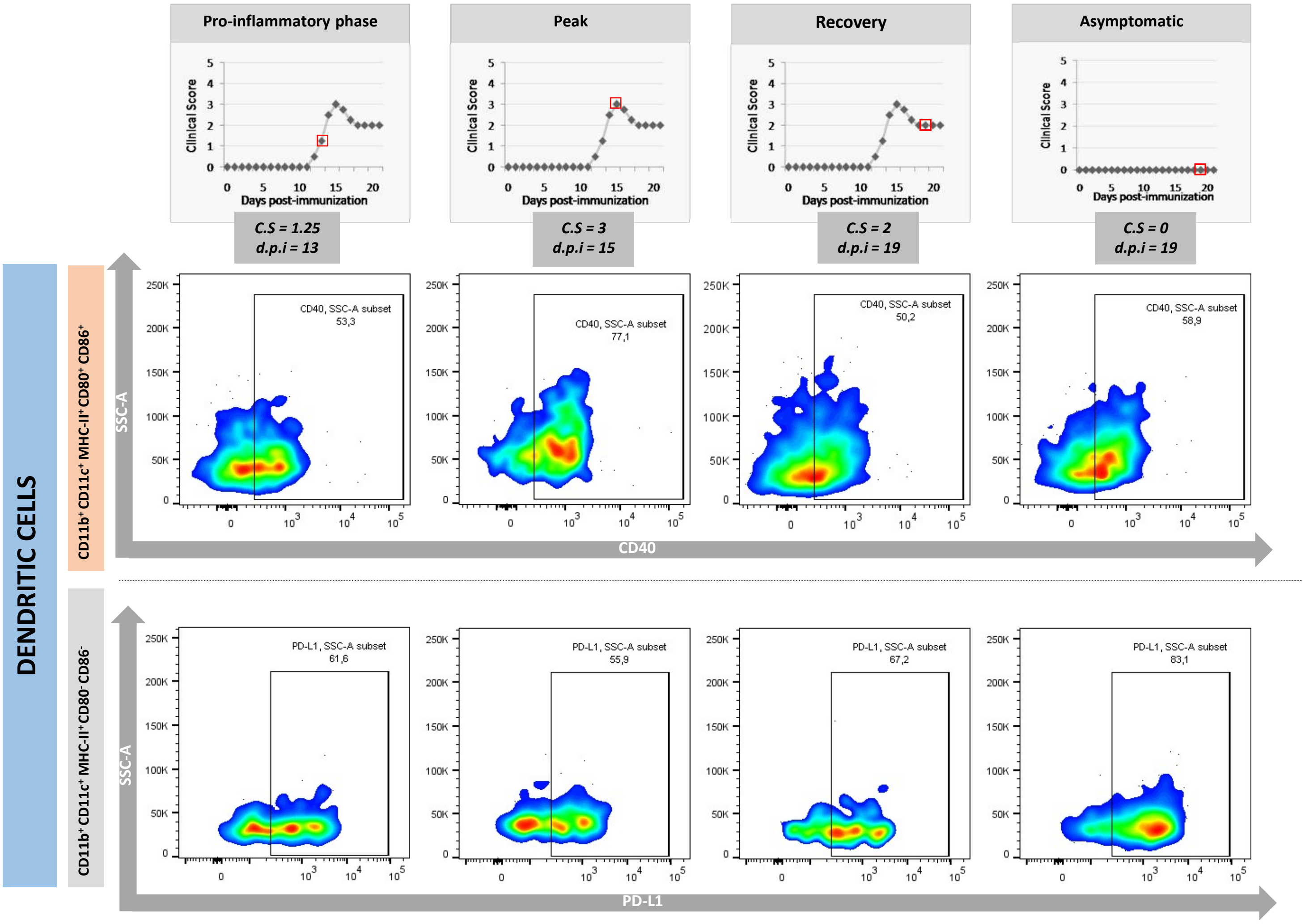

Suppl. 7

Hélie et al., Tissue plasminogen activator worsens experimental autoimmune encephalomyelitis by complementary actions on lymphoid and myeloid cell responses

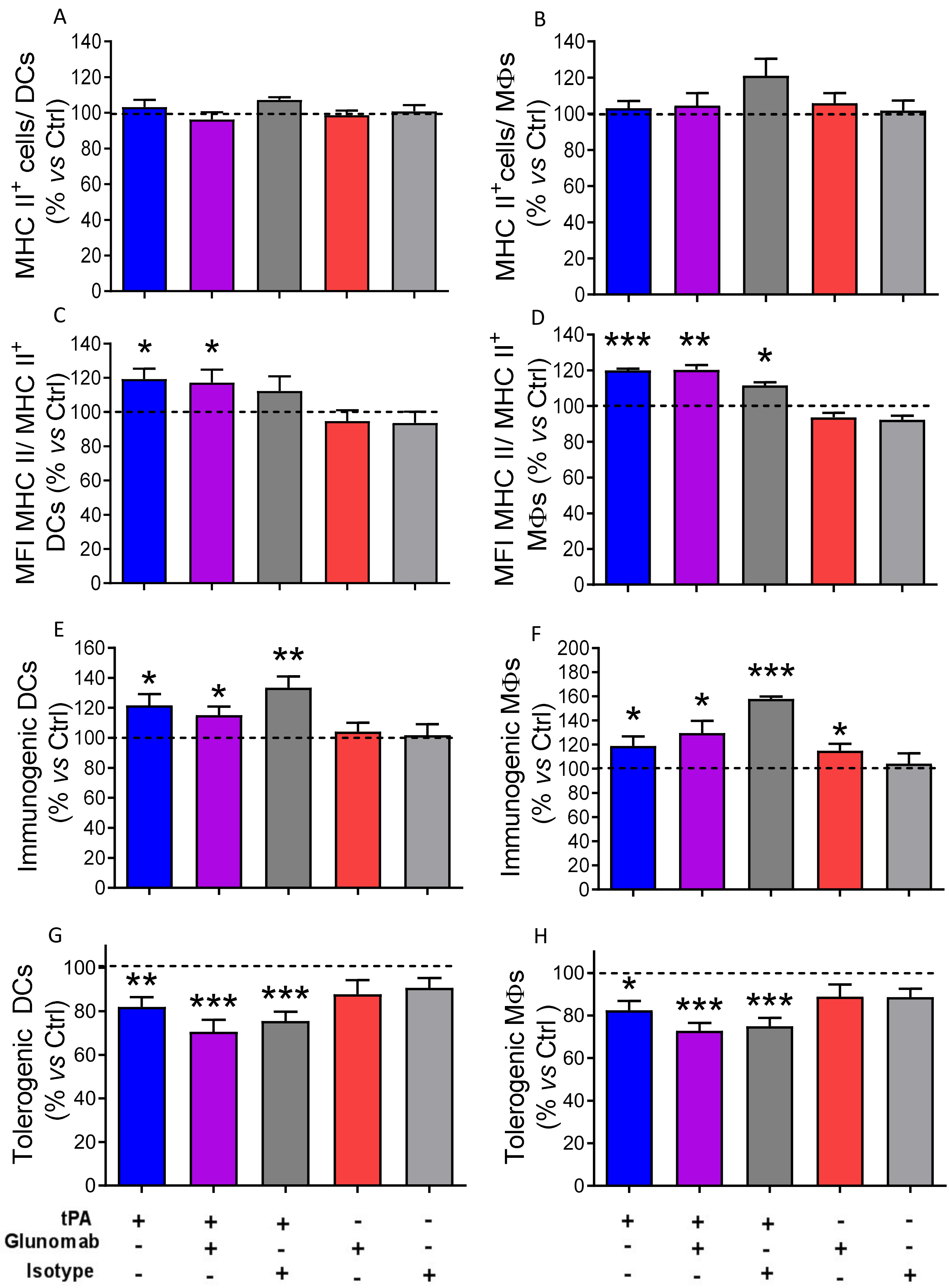

Supplement: Supplementary file 1 — Additional file 1: Suppl Figure 1: Gating strategy and lymphoid cell response after EAE in WT and tPA-/- mice. (A) Representative gating strategy for absolute cell count analysis of leukocyte subsets in spinal cord homogenates (WT mouse, plateau phase of EAE). (B) CD3+ leukocytes were gated for subpopulation analysis after using scattergram gates for viable leukocytes (in A). (C) representative flow cytometry plots for the assessment of intracellular cytokines IFNγ and IL-17 in CD4+ cells after EAE in WT and tPA-/-. Suppl Figure 2: T cell infiltration in the spinal cord of WT and tPA-/- EAE mice. Photomicrographs show representative images (from N=3) of indicated markers in spinal cord tissue samples of WT and tPA-/- mice at the EAE plateau phase (d20±3) from (A) cervical, (B) upper thoracic, and (C) lumbar sacral. Suppl Figure 3: Glunomab does not prevent the effect of tPA on T cells. Splenocytes activated with both anti-CD3ε and anti-CD28 antibodies were treated or not with tPA at 10 μg/mL in presence of Glunomab or the isotypic antibody for 4 days. Proliferation index (%) of (A) CD4+ and (B) CD8+ T cells in the indicated conditions. Results are expressed as mean + SEM (N=3). *P<0.05 vs control #P< 0.05 vs indicated experimental group. Suppl Figure 4: Myeloid response of WT and tPA-/- mice at the plateau phase of EAE. Representative gating strategy for absolute cell count analysis of leukocyte subsets in spinal cord homogenates of WT and tPA-/- mice. CD3- leukocytes were gated for subpopulation analysis after using scattergram gates for viable leukocytes (see Suppl.fig 1A). Suppl Figure 5: Myeloid response to tPA treatment. Representative flow cytometry plots for (A) dendritic cells and (B) macrophages after tPA treatment (0-20 μg/mL). Suppl Figure 6: Dynamic modification of immunogenic and tolerogenic DC phenotype along the EAE clinical course. CD40 is highly present at the moment of maximum affectation and decreased when symptoms partially recover. PD-L1 is incr [file 12974_2021_2102_MOESM1_ESM.pdf]
