# Supplementary material for: Tissue plasminogen activator worsens experimental autoimmune encephalomyelitis by complementary actions on lymphoid and myeloid cell responses
Source: J Neuroinflammation. 2021 Feb 20;18:52. doi: 10.1186/s12974-021-02102-5 (PMC7897384; doi:10.1186/s12974-021-02102-5)
Supplement: Supplementary file 2 — Additional file 2: Suppl. Table 1: Antibodies used in the present study. Each antibody is indicated with its target antigen, host species, clone number, isotype and commercial reference [file 12974_2021_2102_MOESM2_ESM.docx]

**Suppl. Table 1 : Antibodies used in the present study**. Each antibody is indicated with its target antigen, host species, clone number, isotype and commercial reference

| **Name** | **Clone** | **Isotype** | **Reference** |
| --- | --- | --- | --- |
| Purified Rat Anti-Mouse CD16/CD32 | 2.4G2 | Rat IgG2b, κ | BD Biosciences 553142 |
| BV510 Hamster Anti-Mouse CD3e | 145-2C11 | Armenian Hamster IgG1, κ | BD Biosciences 563024 |
| APC Hamster Anti-Mouse CD3e | 145-2C11 | Armenian Hamster IgG1, κ | BD Biosciences 553066 |
| APC Rat Anti-Mouse CD4 | RM4-5 | Rat DA, IgG2a, κ | BD Biosciences 553051 |
| PE Rat Anti-Mouse CD4 | RM4-5 | Rat DA, IgG2a, κ | BD Biosciences 553049 |
| PE-Cy™7 Rat Anti-Mouse CD8a | 53-6.7 | Rat LOU, IgG2a, κ | BD Biosciences 552877 |
| FITC Rat Anti-Mouse CD8a | 53-6.7 | Rat LOU, IgG2a, κ | BD Biosciences 553031 |
| BV421 Rat Anti-Mouse CD25 | 7D4 | Rat LEW, IgM, κ | BD Biosciences 564571 |
| PE Rat Anti-Mouse Foxp3 | MF23 | Rat IgG2b | BD Biosciences 560408 |
| PE-Cy™7 Rat Anti-Mouse CD45 a | 30-F11 | Rat LOU, IgG2b, κ | BD Biosciences 561868 |
| BV421 Rat Anti-CD11b | M1/70 | Rat DA, IgG2a, κ | BD Biosciences 562605 |
| PerCP-Cy™5.5 Rat Anti-CD11b | M1/70 | Rat DA, IgG2b, κ | BD Biosciences 561114 |
| PE-CF594 anti-mouse CD40 | Clone  3/23 | Rat LOU, IgG2a, κ | BD Biosciences 562847 |
| CD11c Monoclonal Antibody (N418), APC | N418 | Armenian Hamster IgG | eBioscience 17-0114-82 |
| F4/80 Monoclonal Antibody (BM8), eFluor 450 | BM8 | Rat IgG2a, κ | Invitrogen 48-4801-82 |
| MHC Class II (I-A/I-E) Monoclonal Antibody (M5/114.15.2), PE-Cyanine7 | M5/114.15.2 | Rat IgG2b, κ | Invitrogen 25-5321-82 |
| PE/Cyanine7 anti-mouse CD183 (CXCR3) Antibody | CXCR3-173 | Armenian Hamster IgG | BioLegend 126516 |
| Brilliant Violet 421™ anti-mouse CD196 (CCR6) Antibody | 29-2L17 | Armenian Hamster IgG | BioLegend 129818 |
| PE anti-mouse IFN-γ Antibody | XMG1.2 | Rat IgG1, κ | BioLegend 505808 |
| FITC anti-mouse IL-17A Antibody | TC11-18H10.1 | Rat IgG1, κ | BioLegend 1506907 |
| BV421 anti-mouse PD-L1 | 10F.9G2 | Rat IgG2b, κ | Biolegend 124315 |
|  |  |  |  |
